# A Systematic Approach to Pair Secretory Cargo Receptors with Their Cargo Suggests a Mechanism for Cargo Selection by Erv14

**DOI:** 10.1371/journal.pbio.1001329

**Published:** 2012-05-22

**Authors:** Yonatan Herzig, Hayley J. Sharpe, Yael Elbaz, Sean Munro, Maya Schuldiner

**Affiliations:** 1 Department of Molecular Genetics, Weizmann Institute of Science, Rehovot, Israel; 2 MRC Laboratory of Molecular Biology, Cambridge, United Kingdom; The Scripps Research Institute, United States of America

## Abstract

The endoplasmic reticulum (ER) is the site of synthesis of secreted and membrane proteins. To exit the ER, proteins are packaged into COPII vesicles through direct interaction with the COPII coat or aided by specific cargo receptors. Despite the fundamental role of such cargo receptors in protein traffic, only a few have been identified; their cargo spectrum is unknown and the signals they recognize remain poorly understood. We present here an approach we term “PAIRS” (pairing analysis of cargo receptors), which combines systematic genetic manipulations of yeast with automated microscopy screening, to map the spectrum of cargo for a known receptor or to uncover a novel receptor for a particular cargo. Using PAIRS we followed the fate of ∼150 cargos on the background of mutations in nine putative cargo receptors and identified novel cargo for most of these receptors. Deletion of the Erv14 cargo receptor affected the widest range of cargo. Erv14 substrates have a wide array of functions and structures; however, they are all membrane-spanning proteins of the late secretory pathway or plasma membrane. Proteins residing in these organelles have longer transmembrane domains (TMDs). Detailed examination of one cargo supported the hypothesis that Erv14 dependency reflects the length rather than the sequence of the TMD. The PAIRS approach allowed us to uncover new cargo for known cargo receptors and to obtain an unbiased look at specificity in cargo selection. Obtaining the spectrum of cargo for a cargo receptor allows a novel perspective on its mode of action. The rules that appear to guide Erv14 substrate recognition suggest that sorting of membrane proteins at multiple points in the secretory pathway could depend on the physical properties of TMDs. Such a mechanism would allow diverse proteins to utilize a few receptors without the constraints of evolving location-specific sorting motifs.

## Abstract 2

A systematic approach to visualize proteins exiting the endoplasmic reticulum paired with their cargo receptors identifies novel cargo for known receptors and reveals the mechanism of one conserved receptor, Erv14.

## Author Summary

All cells sense their environment, respond to it, and communicate with neighboring cells. To perform these functions, cells use an impressive array of proteins that they display on their surface membranes and secrete into their external environment. Newly synthesized proteins destined for the surface of nucleated cells, or to be secreted into the environment must enter the secretory pathway through the endoplasmic reticulum. Those that reside there remain behind, but most leave for their next destination as cargo proteins in lipid vesicles. To be packaged into vesicles, many of them require a “cargo receptor,” which recognizes and tethers specific cargo proteins in the vesicles. Our study takes a systematic approach to identify the range of cargo proteins that bind to each of the known receptors in yeast. By using this approach, we both discover new cargo for known cargo receptors and delineate the rule that governs cargo selection for one cargo receptor, Erv14. Thus, our study demonstrates a novel approach to identify the cargo for any receptor or to discover new cargo receptors.

## Introduction

The endoplasmic reticulum (ER) is the entry site into the secretory pathway, responsible for the folding, maturation, and trafficking of all secreted, membrane-bound, and secretory pathway resident proteins. Once folded, the proteins exit the ER as cargo within COPII-coated vesicles that bud from ER exit sites [1],[2]. Active concentration of proteins into the vesicles [3]–[6] occurs either by direct interaction with the Sec23 and Sec24 subunits of the COPII coat or else are mediated through a diverse group of proteins that function as “adaptors” and have been termed cargo receptors [4],[7]. Cargo receptors allow sorting of cargo that cannot directly bind Sec23/24, or cargo whose exit requires quality control or regulation [8],[9].

The prevalent way to identify cargo for a cargo receptor entails testing selected individual proteins in transport assays or in vitro COPII budding reactions, as was utilized to pair glycosylphosphatidylinositol (GPI)-anchored proteins with their cargo receptors—the p24 family of proteins [10]–[12]. Because of the complexity of these approaches, only a few additional cargo receptors have since been identified (Table S1). Moreover, despite their important function in ER exit and their potential for regulating the flow of traffic in the entire secretory pathway, there is still little information about the entire spectrum of cargos for a given cargo receptor or what defines its cargo specificity. Importantly, no attempt has yet been made to pair large sets of possible cargos with their cargo receptors in a manner that is systematic and unbiased. The lack of systematic data has hindered the identification of the determinants shared by specific sets of cargo that allow their recognition by a particular cargo receptor. Identification of such determinants might also shed light on the purpose and mechanism of action by which a given cargo receptor operates.

Here we describe a systematic approach that aims to complement the traditional methods of cargo and cargo receptor discovery which we call “PAIRS” (pairing analysis of cargo receptors). The PAIRS approach utilizes robotic methodologies to genetically manipulate *Saccharomyces cerevisiae* libraries containing green fluorescent protein (GFP)-tagged cargo [13]–[15], followed by automated microscopy to identify mutated backgrounds that cause ER retention of cargo. Using PAIRS we have increased the number of known cargos for a set of nine cargo receptors. Since our approach probes a large set of proteins for their receptor requirements it defines both groups that are dependent and that are independent of any given cargo receptor. Combined, this should help to define the rules of specificity for each of the cargo receptors. We demonstrate the utility of this approach by using the set of cargo uncovered for the cargo receptor Erv14 to formulate a hypothesis on its mode of substrate recognition. The large group of cargo that require Erv14 as their cargo receptor do not share a detectable functional similarity or sequence motif. However, all identified cargo resides in late secretory pathway membranes that are populated with proteins of longer transmembrane domains (TMDs) than TMDs of ER resident proteins [16]. This raises the hypothesis that cargo specificity of Erv14 is determined by TMD length. Following up on one substrate, Mid2, we show this to indeed be the case. Thus Erv14 may be able to recognize a diversity of cargo by recognizing a shared physical property rather than a specific sequence. This also suggests a resolution for conflicting findings on the effect of TMD length on protein retention in the ER or Golgi [17]–[22].

## Results

### The PAIRS Methodology

To pair as many cargo proteins as possible with their corresponding cargo receptors in a systematic, non-biased approach, we devised a methodology we call PAIRS. PAIRS is based on the idea that when a cargo receptor is missing, then its cargo accumulates in the ER and that this can be visualized by using fluorescently tagged cargo.

**Figure 1:**
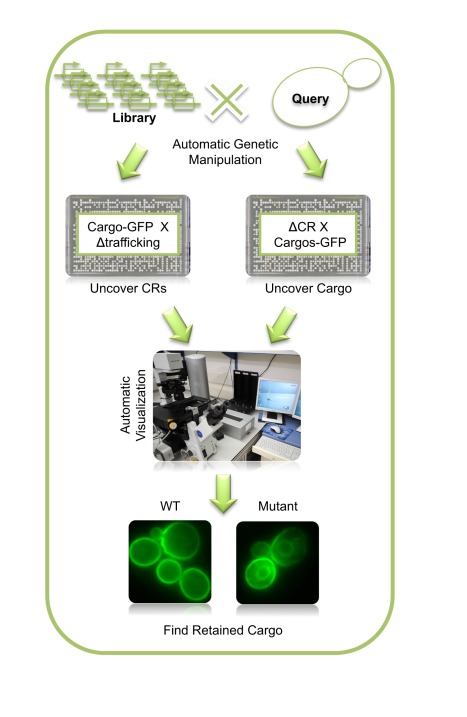
PAIRS: a systematic approach for identification of cargo for a given cargo receptor. Libraries containing deletion in trafficking-related proteins or secretory pathway proteins tagged with GFP were assembled. Using the SGA methodology they were crossed against query strains carrying either a cargo tagged with GFP or a deletion in one of nine different cargo receptors with the assistance of automated cell manipulation techniques. Libraries were then automatically imaged for GFP localization followed by manual inspection of the images, enabling detection of ER retention due to a given deletion.

The PAIRS approach can be used for two purposes. First, it can be used to uncover the cargo receptor for a specific cargo of interest by expressing that specific cargo fused to GFP on the background of mutations in trafficking-related proteins. Second, it can be used to uncover the spectrum of cargo for a putative cargo receptor by visualizing a large number of strains with various GFP-tagged cargo on the background of mutations in that cargo receptor. The approach relies on systematic creation of genetically modified strains using the synthetic genetic array (SGA) methodology [13],[14],[23], which is followed by acquisition of fluorescent images of all strains using a high-throughput automated microscopy platform. Finally, manual examination of the resulting images uncovers strains in which the mutation causes ER retention of cargo, implying a cargo receptor/cargo pair (Figure 1).

### Uncovering the Cargo Receptor for a Given Cargo Using PAIRS

**Figure 2:**
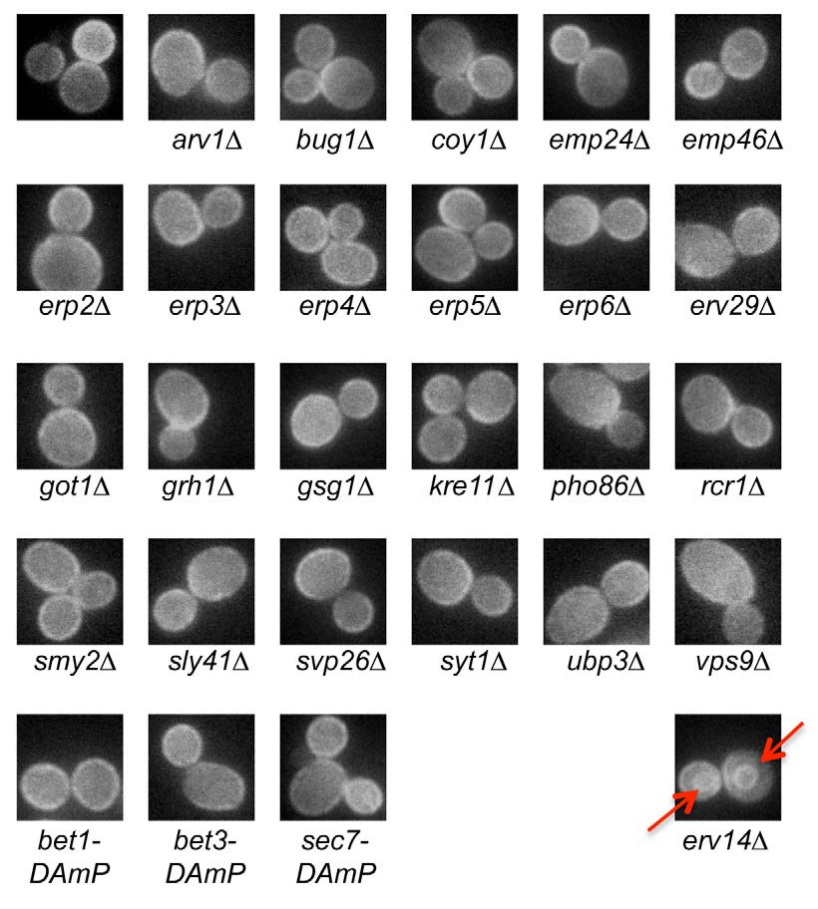
PAIRS can uncover the cargo receptor for a given cargo. Deletions of 46 proteins involved in ER to Golgi trafficking (representative examples shown, for a full list see Table S6) were screened during stationary phase for their effect on Tpo4-GFP localization. Only Δ*erv14* caused ER retention, suggesting that it functions as the cargo receptor for Tpo4-GFP (altered localization is marked with a red arrow). Pictures were taken at a magnification of 60×.

To determine whether our methodology can indeed facilitate the identification of a cargo receptor for an arbitrary cargo, we chose Tpo4, a plasma membrane multidrug transporter involved in polyamine transport, whose ER exit had not been shown to rely on a particular cargo receptor. We first created an SGA compatible query strain expressing Tpo4-GFP from its endogenous promoter. We then collected strains of mutants in trafficking related proteins from either the deletion library (for non-essential genes) [11], or from the decreased abundance by mRNA perturbation (DAmP) library (for hypomorphic alleles of essential genes) [24] (for a full list of strains used and the proposed function of their corresponding protein see Table S6). Using the SGA approach [14], we crossed the Tpo4-GFP into the mutant library creating a new library of haploid yeast strains each expressing Tpo4-GFP on the background of a mutation in a single gene. Visualization of these strains demonstrated that all but one of the strains did not alter Tpo4-GFP's localization (Figure 2). Only the Δ*erv14* strain displayed ER accumulation of Tpo4-GFP (red arrows in Figure 2). Although Erv14 is a known cargo receptor [25]–[27], it has not been previously implicated in trafficking of Tpo4. Our analysis suggests that Erv14 is the cargo receptor for Tpo4 and demonstrates that the PAIRS methodology can be used to find a cargo receptor for a given cargo of interest.

### Using PAIRS to Uncover the Spectrum of Cargos for a Given Cargo Receptor

We next wanted to utilize the PAIRS methodology to map the spectrum of cargos for a cargo receptor of interest. We therefore created nine query strains, each carrying a deletion or a DAmP hypomorphic allele of a putative cargo receptor: Δ*erv14,* Δ*erv15, Δerv26,* Δ*erv29,* Δ*emp24,* Δ*emp47,* Δ*gsf2,* Δ*chs7*, and *Shr3-DAmP*. To generate a library of GFP-tagged cargo we used the Yeast GFP Fusion Localization Database to identify fusion proteins that were reported to reside in post-ER compartments (Golgi, puncta, vacuole, or cell periphery) [15]. These were filtered to remove all those without TMDs or a signal peptide giving rise to 157 strains. The strains and controls were assembled and all of these were crossed into each of the nine query strains thus generating nine new libraries of GFP-tagged cargo proteins, each lacking an individual putative receptor.

**Figure 3:**
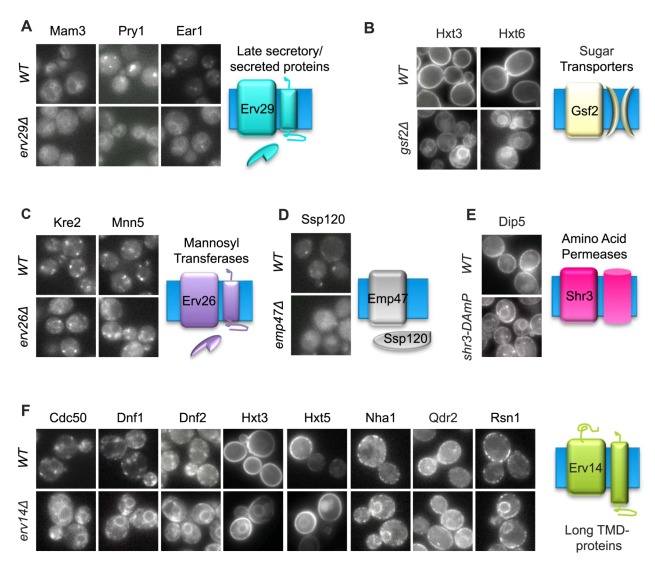
PAIRS allows pairing of secretory pathway cargo with their respective cargo receptors. Mutations in nine cargo receptors were studied for their effect on cargo retention. Shown are the proteins that displayed ER retention phenotype on the background of (A) mutations in Δ*erv29*, (B) Δ*gsf2*, (C) Δ*erv26*, (D) Δ*emp47*, (E) *shr3-DAmP* relative to a control (wild-type [WT]) strain during logarithmic growth. Shown for each is a cartoon hypothesizing the common denominator of its respective cargo. (F) Deletion of Erv14 caused widespread ER retention. Shown are localizations of eight GFP-tagged proteins during logarithmic growth in control strains (WT) relative to Δ*erv14.* For a full list, see Figure S2A. Pictures were taken at a magnification of 60×.

Inspection of the strains showed that the majority of cargos (126 out of 157) managed to exit the ER in all deletion backgrounds, suggesting that they do not solely rely on any one of the nine cargo receptors studied here for their ER exit. This suggests that most proteins can either bind the COPII coat directly, depend on redundant mechanisms for ER exit, rely on as yet undiscovered cargo receptors, or that they are exported out of the ER by spontaneous “bulk flow.” However, for all but one cargo receptor, Erv15, we could find at least one cargo that depended on it. The annotation of Erv15 stems from its high homology to the cargo receptor Erv14 (63%), and it appears to be required to augment the activity of Erv14 in transporting particular cargo in sporulating cells but not under normal growth conditions [25],[26]. The full set of novel cargos found for each of the other seven cargo receptors is shown in Figure 3 (for previously characterized cargo that were verified by the screen see Figure S1). It appears that there is never a complete blockage of ER exit; this may simply reflect the proteins leaking out of the ER once they have accumulated to high levels, but there may also be some functional redundancy in the ER exit machinery.

Since this is the first time that all cargo receptors have been studied in the same system and under the same conditions in a systematic manner, the spectrum of cargo uncovered for each cargo receptor could also be used to start defining the functional rules guiding the recognition mode. For example, all cargo for Erv26 comprised of Golgi-localized mannosyltransferases (Figure 3C) as had previously been suggested [28],[29]. However Erv26 seems to be specific to a subset of this functional group as several additional mannosyltransferases (Mnn1-GFP, Mnn11-GFP, Mnn10-GFP, Anp1-GFP, and Hoc1-GFP) did not accumulate in the ER in this background (unpublished data). Another example for specificity is our finding of only a single novel cargo for Shr3 and the identity of this cargo as an amino acid permease (Figure 3E) as are all previously identified cargo, supporting the notion that Shr3 is a dedicated cargo receptor for amino acid permeases [8],[9],[30],[31]. A similar picture emerges for Gsf2 whose novel cargo all fall into the same functional category of sugar transporters (Figure 3B) as reported previously [32]. Moreover, the sugar transporter Hxt2-GFP previously shown to be independent of Gsf2, is indeed properly localized to the plasma membrane (unpublished data), supporting the notion that Gsf2 is involved in exit of only particular sugar transporters from the ER. Other cases are less clear, such as the three seemingly unrelated cargo that we uncovered for Erv29 (Figure 3A). Previous reports identified three soluble proteins as requiring Erv29 for efficient ER exit (PrA, CPY, and α-factor) [33],[34], and although one of the new proteins is a soluble protein (Pry1), two others (Ear1 and Mam3) are membrane proteins of the vacuole or endosome.

### Erv14 Is Required for the ER Exit of a Diverse Range of Proteins

Perhaps the most striking finding is the large number of proteins that require Erv14 for efficient ER exit. Erv14 was identified as being enriched in COPII vesicles and shown initially to be required for the ER exit of the plasma membrane protein Axl2 [26]. Recent work has shown that mutants lacking Erv14 also show ER accumulation of the proteins Sma2 [25], Mid2, Gap1, Hxt1, and Hxt2 [35]. Our PAIRS approach identified that Erv14 is required for the ER exit of 32% of the plasma membrane proteins checked (18 of 57) (Figures 3F and S2A and S2B). Among these proteins are permeases (e.g., Mep2-GFP), transporters (e.g., Hxt2-GFP and Nha1-GFP), multidrug transporters (e.g., Snq2-GFP and Tpo4-GFP), lipid flippases (e.g., Cdc50-GFP and Dnf1-GFP), eisosome components (Sur7-GFP), and proteins involved in cell polarity or cell wall regulation (e.g., Mid2-GFP and Axl2-GFP). Some have a single TMD whilst others are polytopic with up to 12 TMDs. Hence there is no obvious functional or structural similarity between the proteins affected by Erv14.

Consistent with previous work, Erv14 was not required for ER exit of soluble proteins and non-conventional membrane tethered proteins such as GPI-anchored and tail-anchored proteins (Figure S3) [26],[35]. To strengthen the predictions made by our PAIRS methodology we analyzed the physical interactors of Erv14 under the assumption that direct cargo should physically interact with its cargo receptor. To this end, we immunoprecipitated HA-tagged Erv14 (which completely retains the function of the endogenous Erv14 [36]) from microsomes and analyzed the precipitated proteins by mass spectrometry. Using this approach we could corroborate eight out of the 23 cargo predicted by PAIRS as physically interacting (Figure S4A). We also found five interacting proteins that could be cargo; however, they were not examined in our original screen because of mislocalization of the C-terminal tagged fusion. To verify these proteins as cargo we made strains expressing N-terminal GFP fusion proteins and found that two of them are indeed retained in the ER in Δ*erv14* (Figure S4B).

Live cell imaging confirmed that Erv14's absence decreased the kinetics of ER exit of predicted cargo (Figure S5), raising the question of how it could accelerate the ER exit of such a defined set of diverse proteins. We performed in-depth sequence analysis of Erv14 cargo but could not uncover any identifiable sequence motifs (unpublished data). However, the fact that all cargos of Erv14 are membrane proteins destined to reside in the membranes of the late secretory pathway suggested that inherent characteristics of the membrane-spanning region might be responsible for the recruitment of Erv14. Indeed, a comprehensive comparison of TMDs of bitopic proteins from different compartments has shown that TMDs from post-Golgi compartments are significantly longer, suggesting that the bilayer is thicker [16]. Thus a larger hydrophobic portion, adapted for the apparently thicker bilayer of the plasma membrane, may be the trait that determines potential cargo for Erv14. We thus investigated the dependence of ER exit of an Erv14-regulated cargo on its TMD length.

### Creating a Model Substrate to Characterize the Effect of TMD Length on Erv14-Dependent ER Exit

**Figure 4:**
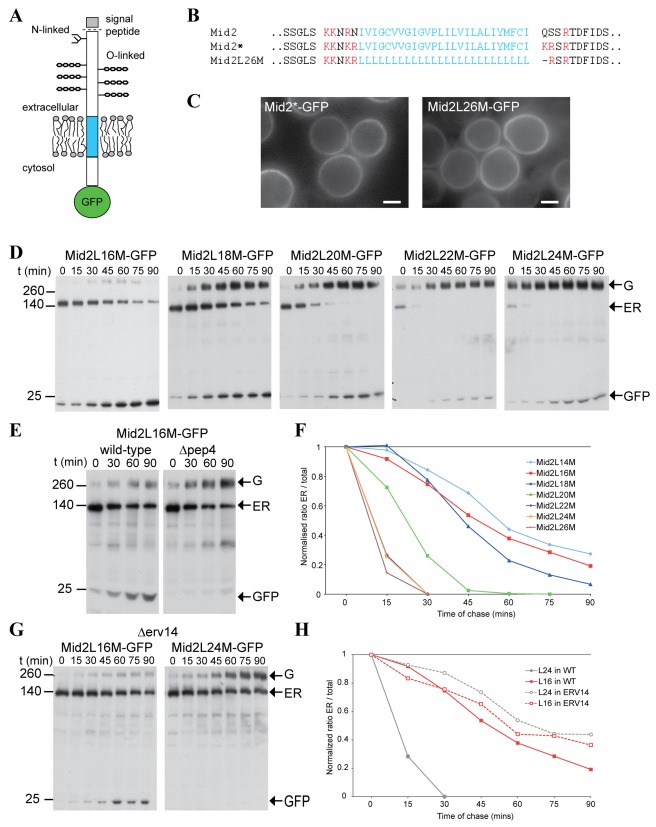
The rate of exit from the ER of polyleucine TMD chimeras of Mid2-GFP depends on TMD length and the presence of Erv14. (A) Mid2 is a type I membrane protein with an exoplasmic N terminus and cytosolic C terminus. Mid2 has a single N-linked glycan and extensive O-linked glycans. (B) Sequence of the TMD region of Mid2 and representatives of the polyleucine variants examined in this study. In Mid2* the flanking residues are modified to insert cloning sites and place basic charges at either end of the TMD. Residues 215–260 of pre-Mid2 shown. (C) Wide-field fluorescent micrographs of yeast (BY4741) expressing the indicated forms of Mid2 from a GAL CEN plasmid and induced with galactose (90 min) and chased for 40 min in glucose. Scale bars 2 µm. (D) Anti-GFP immunoblots of whole cell lysates from yeast expressing the indicated polyleucine TMD variants of Mid2-GFP. The variants were expressed under the control of the *GAL1* promoter from constructs integrated at the MID2 locus. The cells were induced with galactose for 2 h, and then harvested at the times indicated after replacing the medium with that containing 2% glucose. The arrows indicate the ER form (ER) and Golgi modified form (G) of Mid2 [37], and free GFP. These blots are representative of four independent experiments, with similar results observed with a Mid2 length series with a slightly different flanking residues (Figure S5B). (E) As (D) except that one yeast strain lacks the vacuolar protease Pep4. (F) Graph of ER exit of the polyleucine variants shown in (D) and in Figure S5A. The proportion of the total material (ER, Golgi-modified, and free GFP) that is ER form is plotted against the time in glucose. The graphs are normalized such that all start at 1.0 at *t* = 0 min. (G) Anti-GFP immunoblots of whole cell lysates from yeast expressing long (L24) and short (L16) polyleucine TMD variants of Mid2-GFP in cells lacking Erv14. The cells were induced with galactose for 2 h and harvested as in (D). The arrows indicate the ER form (ER) and Golgi modified form (G) of Mid2, and free GFP. The rate of exit from the ER of the L24 form is greatly slowed (compare to (D)). The results are representative of three independent experiments. (H) Graph of ER exit of the constructs shown in (G), as calculated for (F).

To assay the effect of TMD length on protein sorting by Erv14 we used the plasma membrane cargo protein Mid2 as a reporter. Mid2 is a non-essential type I membrane protein with a signal peptide and a single, 26–amino acid-long, TMD (Figure 4A). One advantage of using Mid2 is that its maturation along the secretory pathway can be monitored owing to the presence of luminal modifications by a single N-linked glycan and multiple O-linked glycans. Since the extension of the O-linked glycans occurs in the Golgi and results in reduced mobility on SDS gels, this can be used to assay the extent of ER exit [37]. To remove any possible interference that may stem from specific sequences in the TMD we replaced the endogenous TMD with a stretch of 26 leucines (Mid2L26M). The residues at either end of the TMD were modified to be basic to provide sharp ends to the hydrophobic region (Figure 4B), and because basic residues are the most common charged residues at both the cytoplasmic and luminal ends of the TMDs of yeast plasma membrane proteins [16]. To ascertain that these changes did not alter the basic cargo properties of Mid2, we expressed a GFP-tagged form of Mid2L26M in yeast and observed that it localizes to the plasma membrane in a manner identical to the wild-type protein (Figure 4C). This finding indicates that our synthetic reporter is correctly localized and that Mid2 does not require specific motifs within its TMD for its trafficking through the secretory pathway.

### The Length of the TMD Affects the Location of Mid2 in an Erv14-Dependent Manner

We next generated variants of Mid2 in which the polyleucine TMD was shortened in increments of two residues to give a set of variants spanning the range of 14–26 residues. To examine the trafficking of these variants, each one was expressed under the control of a galactose-inducible, glucose-repressible, promoter. By inducing transcription with galactose for 90–120 min followed by termination upon return to glucose, it was possible to generate a pulse of protein whose progress through the secretory pathway could be followed by blotting and microscopy.

When we compared the TMD-length variants by blotting we found that they behaved differently (Figures 4D and S6A). The majority of the longest TMD form was exported from the ER even during the pulse of induction, and the remaining material was rapidly matured during the chase. However, as TMDs became shorter in the variants, the ER form took a longer time to disappear. Similar results were obtained with an independent set of TMD variants in which the polyleucine stretch was flanked by a tryptophan at either end, a residue sometimes enriched in this position (Figure S6B). In fact, in the shorter TMD variants we could not observe Golgi forms accumulating but instead we observed an accumulation of a band corresponding to free GFP. This free GFP likely reflects degradation of the short TMD variants in the vacuole, as it is not present in a strain expressing the 16-leucine variant (Mid2L16M) and lacking the vacuolar hydrolase Pep4 (Figure 4E). Therefore we assume that once out of the ER and in the Golgi, the shorter TMD variants are directed to the vacuole and degraded, whereas the longer TMD forms are trafficked correctly to the plasma membrane. Thus, the steady state pool of the Golgi-modified, but not yet degraded, form of the shorter variants will be low. Quantitation of the ER form confirmed that the longer TMD variants exited the ER much more rapidly, with 20 leucines being the point of transition (Figure 4F).

We next asked what effect TMD length has on Erv14-dependent exit. By repeating the pulse chase experiments in a Δ*erv14* strain we found that the ER exit of the long form of Mid2 was drastically slowed down, demonstrating a dependence on Erv14 (Figure 4G). However, we could not observe any change in the slow exit rate of the short TMD form Mid2L16M, indicating that this shorter form exits the ER in an Erv14-independent manner (Figure 4H). Taken together with the fact that the polyleucine TMD did not abolish Erv14 dependence of Mid2L24M, this strongly supports the idea that the length of the TMD and not its sequence are a determinant of Erv14-mediated sorting.

### ER Exit of Mid2 Variants with Shorter TMDs Can Be Rescued by Adding a COPII-Binding Motif

**Figure 5:**
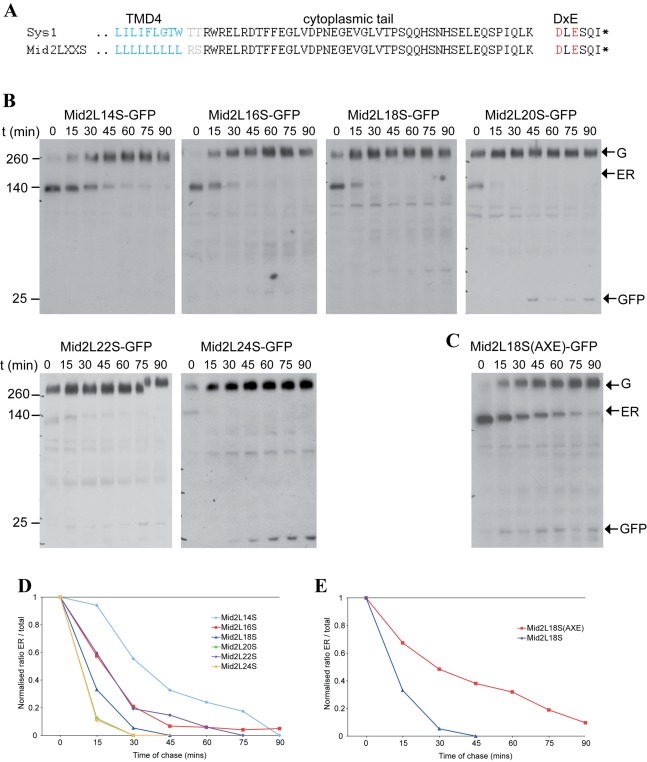
The cytoplasmic tail of Sys1 accelerates the ER exit of short TMD variants of Mid2. (A) The C-terminal cytoplasmic tail of the polytopic membrane protein Sys1. The end of the last of its four TMDs is shown, along with the DXE motif that binds COPII [38]. Also shown is the structure of the Mid2 polyleucine variants with the Mid2 tail replaced with that of Sys1 (see also Figure 4B). (B) Anti-GFP immunoblots of whole cell lysates from yeast expressing the indicated polyleucine TMD variants of Mid2-GFP with tail replaced with that of Sys1. The variants were expressed under the control of the *GAL1* promoter from constructs integrated at the MID2 locus. The cells were induced with galactose for 2 h, and then harvested at the times indicated after replacing the medium with that containing 2% glucose. The arrows indicated the ER form (ER) and Golgi modified form (G) of Mid2-GFP, and free GFP. For reasons that are unclear we could not obtain cells expressing the L26 variant at the same levels as the other constructs and so it was omitted. The results are representative of three independent experiments. (C) As Mid2L18S-GFP in (B), except that the DXE motif is mutated to AXE. (D and E) Graphs of ER exit of the constructs shown in (B and C), as calculated for Figure 4F.

The above results are consistent with the idea that Erv14 selectively acts as a cargo receptor on proteins with a long TMD. However it is also possible that a short TMD acts as an ER retention signal by discouraging entry into COPII vesicles or ER exit domains, and hence this effect over-rides the ability of Erv14 to extract the cargo from the ER. To investigate this possibility we asked whether the short TMD forms of Mid2 could exit more rapidly if directed to COPII vesicles by a different mechanism. Thus we created new versions of the Mid2 reporter that were fused with the cytoplasmic tail of the Golgi-localized Sys1 protein that contains a DXE motif for direct binding to Sec24 (Figure 5A) [7],[38],[39].

When these constructs were expressed in wild-type cells we found that the ER exit of the short forms was significantly accelerated in comparison to the form that lacked the DXE motif and was now closer to the rate of the longer TMDs (Figure 5B and 5D). In addition, we observed a reduction in the levels of free GFP, indicating that once the constructs had left the ER, they were no longer rapidly degraded. When the DXE motif in the Sys1 tail was mutated to AXE the rate of ER exit was reduced, confirming that the effect is mediated by COPII binding (Figure 5C and 5E). Interestingly, the stabilization of the shorter TMD variants (i.e., the reduction in accumulation of free GFP during the chase) conferred by the Sys1 tail was retained even after DXE was mutated, suggesting that other sequences in the Sys1 tail are responsible for this effect. Taken together, these results indicate that the reduction in ER exit rate seen upon TMD shortening is a feature of an Erv14-dependent cargo, but not a feature of a cargo protein that is concentrated in COPII vesicles by other mechanisms. This suggests that Erv14 specifically directs the ER exit of proteins with longer TMDs.

### A General Role for TMD Length-Dependent Sorting

**Figure 6:**
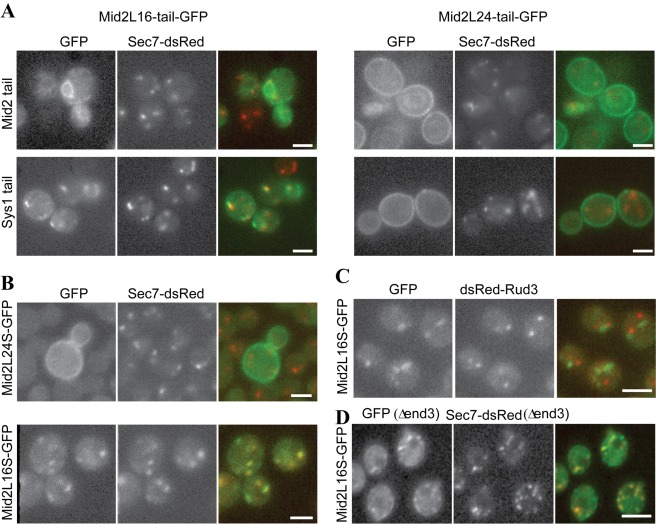
TMD length can affect protein sorting also at the trans Golgi. (A) Wide-field fluorescent micrographs of live yeast expressing the indicated Mid2-GFP with either 16 or 24 leucines in the TMD and the Mid2 or the Sys1 cytoplasmic tail as indicated. The strains are the same as those in Figures 4 and 6 with the chimeras expressed from the *GAL1* promoter and imaged after 90 min of galactose induction. The cells are also expressing Sec7-dsRed to label the trans-Golgi. Scale bars 2 µm. (B) Wide-field fluorescent micrographs of live yeast expressing Mid2-GFP with either 16 or 24 leucines in the TMD and the Sys1 cytoplasmic tail. The chimeras are constitutively expressed from the *MID2* promoter by integration at the endogenous MID2 locus in the same Sec7-DsRed strain as in (A). Mid2L16S-GFP was imaged using a beam splitter. Scale bars 2 µm. (C) Mid2 with a 16 leucine TMD as in (B) except compared to the cis Golgi marker Rud3. Scale bar 2 µm. (D) Wide-field fluorescent micrographs of live yeast constitutively expressing Mid2-GFP with a 16 leucine TMD and a Sys1 cytoplasmic tail as in Figure 7A, except the strain lacked the End3 gene required for endocytosis. The construct is still associated with the Golgi labeled with Sec7-DsRed. Golgi cisternae are not stacked in yeast and so the two proteins are likely to be in the same cisternae, but they only partially overlap, perhaps reflecting one or both being non-uniformly distributed within the cisternae. Scale bar 1 µm.

In the above experiments we found that the Sys1 tail enabled all of the polyleucine TMD variants to efficiently exit the ER. This allowed us to investigate the effect of TMD length on later trafficking steps without complications from variations in ER exit rate. We thus examined the distribution of the different TMD length forms at the end of the galactose pulse (Figure 6A). As expected, the longer TMDs showed predominantly plasma membrane staining. However, the shorter TMD variants accumulated in intracellular puncta that were also labeled with the late Golgi protein Sec7 (Figure 6A).

This finding suggests that TMD length affected Golgi exit as well as ER exit. To confirm that this difference was not due to kinetic effects, we re-examined the distribution of TMD variants expressed constitutively under the control of the endogenous Mid2 promoter. As observed for the galactose-induced versions, the longer TMD variants show a clear plasma membrane localization, but the variants with TMDs of less than 22 residues again accumulated in puncta with little if any cell surface staining (Figure 6B). These puncta co-localized with the late Golgi marker Sec7, but not with the early Golgi marker Rud3, confirming that the protein was accumulating in trans Golgi compartments (Figure 6B and 6C). The same Golgi accumulation of shorter TMDs was observed in cells lacking the End3 protein that is required for endocytosis [40], negating the possibility that the shorter TMD variants are simply travelling to the surface and being more rapidly endocytosed (Figure 6D) [41].

**Figure 7:**
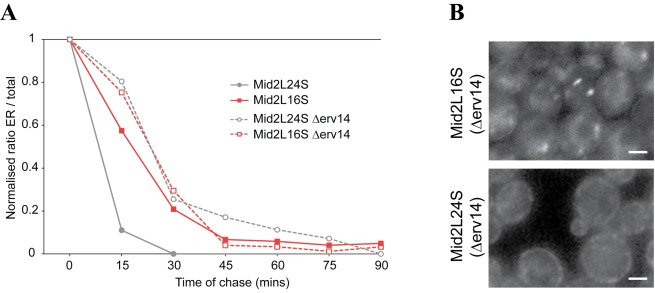
TMD length dependent localization to the trans Golgi can be independent of endocytosis or differential ER exit rate. (A) ER exit rates of the Mid2-GFP with either 16 or 24 leucines in the TMD and the Sys1 cytoplasmic tail in wild-type or a strain lacking Erv14. Cells were induced with galactose and chased as in Figure 5. Removal of Erv14 results in there being no difference in the ER exit rate of the 16 and 24 leucine TMD variants. (B) Wide-field fluorescent micrographs of live yeast cells expressing Mid2-GFP with either 16 or 24 leucines in the TMD and the Sys1 cytoplasmic tail. The chimeras are constitutively expressed from the *MID2* promoter by integration at the endogenous *MID2* locus in strain lacking Erv14. Despite similar ER exit rates the two chimeras have a different intracellular distribution. Scale bars 1 µm.

To determine whether this TMD length-dependent sorting at the Golgi also required Erv14 we expressed the 16 and 24 leucine constructs with Sys1 tails in Δ*erv14.* Both variants displayed the same ER exit rates; however, they still differed in location (Figure 7A and 7B). This indicates that the difference in localization was not dependent on any residual differences in ER exit rate conferred by the Erv14 protein, or by Erv14 chaperoning the protein through the Golgi and having a second role at Golgi exit. Taken together, these results demonstrate that the length of the TMD can determine the rate at which Mid2 leaves the Golgi to travel to the plasma membrane and, unlike the effect of TMD length on ER exit rate, this sorting does not require Erv14.

## Discussion

The PAIRS methodology aims at pairing cargos with their cargo receptors and to elaborate on the existing body of knowledge on cargo extraction from the ER. Our analysis did not include all possible cargos since the proteins in the GFP library are all C-terminally tagged and under their endogenous promoter. This resulted in a number of strains whose proteins were mislocalized due to the tag or had fluorescence levels below detection [15]. This may explain why we could not identify some of the previously recognized cargo/cargo receptor pairs. Despite these caveats, the power of the PAIRS analysis is that it is not biased within the set of pre-selected proteins, allowing us a broad overview of cargo receptors function. This has allowed us to gain insight to the rules governing their specificity and to present the first step towards creation of a cellular “traffickome.”

We demonstrate the value of the PAIRS approach by identifying putative new cargo proteins for most of the known cargo receptors of yeast. This represents 31 of the 157 proteins tested, of which 27 had not been previously linked to a cargo receptor. This is likely to be a slight underestimate of the success rate as some of the proteins stated to have a vacuolar localization in the GFP Database probably reflect ER residents displaced by the tag and hence would not be expected to have a dedicated cargo receptor. One general trend seen across all hits is that removal of a receptor did not result in a complete block of ER exit of its GFP-tagged cargo. This is consistent with previous studies of known cargo/receptor pairs including GPI-anchored proteins [10]. It may be that cargo receptors typically act to accelerate the exit of a particular cargo, and that some bulk flow always occurs, with the volume of this flow increasing for a particular protein if it accumulates in the ER.

The increased knowledge of the range of cargo for each specific cargo receptor should make it easier to generate hypotheses as to what determines the selective recognition of particular cargo by individual cargo receptors. Indeed, examination of the spectrum of cargo relying on the cargo receptor Erv14 suggested that this large and non-homogenous group is recognized on the basis of the length of its TMD. By assessing this hypothesis using one particular cargo, Mid2, we found that TMD length is a major determinant for allowing Erv14 to accelerate exit from the ER. It is still feasible that Erv14 recognizes a more specific motif in Mid2 adjacent to the TMD, and alterations in the length of the TMD affect the position of this region relative to the bilayer and thus prevent Erv14 binding. However, this seems unlikely given the wide range of bitopic and polytopic proteins that are affected when Erv14 is deleted, and their lack of shared sequence motifs. Interestingly, there have been two recent studies reporting that shortening the TMD of a mammalian protein reduces its exit rate from the ER [18],[19]. The mechanism for these effects was not determined, but one reporter used was VSV-G that has been found to depend on the Erv14 paralogue, CNIH4, for normal ER exit [42].

Whether Erv14 enables exit of long TMD-containing proteins from the ER by performing more than just COPII coupling is yet to be uncovered. One option is that it could also act as a chaperone to protect protruding hydrophobic residues on cargo proteins thus enabling them to assume a correct conformation in the shorter ER membranes. Another option is that it sorts long TMD containing proteins into areas of the ER that have thicker membranes thereby enabling their recruitment to vesicles. Regardless, it seems that speed of ER exit may be a major determinant in Erv14's function. In yeast, one of the substrates, Axl2, has been shown to require very rapid ER exit [26], as it must be inserted into the forming yeast bud at a particular point in the cell cycle (*AXL2* mRNA is under cell-cycle control) [43]. One of the substrates for the *Drosophila* paralogue of Erv14, Cornichon, is Gurken, a TGFα-like bitopic protein [44]–[46]. The ability of Gurken to polarize *Drosophila* oocytes depends on its rapid exit from a restricted region of the oocyte ER following translation from a pool of mRNA that is spatially restricted for a short time during development [47],[48]. Indeed, also the action of Cornichon on Gurken requires that the latter has a TMD [47],[49].

Our analysis of the substrate recognition mode for Erv14 reveals that TMD length-dependent sorting may be a more general principle in cellular trafficking than previously appreciated. Using our DxE-containing Mid2 variant we noticed that the TMD variants also underwent a TMD length-dependent sorting in the Golgi apparatus. This is consistent with a previous study examining the effects of lengthening the TMD of the yeast ER protein Ufe1, although this is the first time it has been shown to occur with a homogenous synthetic TMD rather than a native TMD, which may contain additional cryptic sorting motifs [50].

How might length-dependent sorting occur in the Golgi if it does not involve Erv14? One option is that a dedicated cargo receptor exists at this compartment that has not yet been identified. However, an alternative option is that the vesicle composition itself plays a major role in this step with lipids and/or cargo proteins directing a change in bilayer properties [16],[51]–[53].

In summary, our unbiased approach allowed the formulation of a simple hypothesis for the underlying commonality allowing cargo identification by Erv14. Using Mid2 with a synthetic TMD has allowed us to indeed observe such TMD length-dependent steps both in the ER and the Golgi. The notion that TMD length is used by the cell to sort proteins is appealing [51],[53],[54], since many and diverse membrane proteins must be continuously extracted from the ER following synthesis. If these proteins share a generic feature that reflects their normal environment being different to that present in the ER, in this case TMD length, then it would provide a simple means of sorting of many different proteins without the need for specific linear signals.

More generally, the conceptual methodology that we have put forward here could be applied in a wider context to uncover protein localization changes that occur in the absence of any specific gene in the genome. The notion of the “traffickome” could be extended to other trafficking events such as retrograde Golgi to ER traffic, Golgi to plasma membrane traffic, or Golgi to vacuole traffic. Hence, by pairing high-throughput genetic manipulations with a microscopic output it is now possible to study basic questions of specificity and promiscuity in cell biology that have previously been difficult to tackle.

## Materials and Methods

### Media and Growth Conditions

Cultures were grown at 30°C in either rich medium (1% Bacto-yeast extract [BD], 2% Bacto-peptone [BD], and 2% dextrose [Amresco] or synthetic [S] minimal medium [0.67%] yeast nitrogen base without amino acids [Conda Pronadisa)] and 2% dextrose) containing the appropriate supplements for plasmid selection. When necessary, dextrose was replaced by galactose (2%; Amresco) or raffinose (2%; Amresco). For galactose induction, overnight cultures in SD, SD-LEU, or SD–URA cells were diluted 1/10 and grown at 30°C to early log phase in SD or SD–URA medium, then washed and resuspended in 2% galactose-containing SG or SG–URA medium for 2 h. For pulse-chase lysates, the first time point was obtained directly from the galactose culture. Cells were resuspended in glucose-containing medium for chase time points. When needed as selection markers, G418 (200 µg/ml; Calbiochem) or Nourseothricin (Nat) (200 µg/ml WERNER BioAgents) were added. In cases where G418 was required in a SD-based medium, yeast nitrogen base without ammonium sulfate (Conda Pronadisa) was added and supplemented with mono-sodium glutamate (Sigma) as an alternative nitrogen source. Manipulations of plasmid DNA were performed in *Escherichia Coli* strains DH5α and TOP10. A complete list of plasmids used in this study can be found in Table S2.

### Yeast Strains and Strain Construction

All yeast strains in this study are based on the BY4741 laboratory strain [55]. General laboratory strains and strains created in this study are listed in Table S3. Unless otherwise stated, strains harboring a deletion in a specific ORF were taken from the yeast deletion library [11], while strains harboring a hypomorphic allele of an essential gene were taken from the DAmP library [24]. Strains harboring an ORF endogenously tagged with GFP in its C terminus were taken from the yeast GFP library [15]. Genomic modifications and introduction of plasmid DNA were done as previously described [56]. YMS792, YMS793, and YMS954 were created by targeting the *erv14*, *erv15*, and *emp24* genes, respectively, for disruption with the kanR gene with pFA6a-KanMX6 [57]. *MID2* was cloned by PCR in frame with a *GAL1* promoter and monomerized GFP (A207K) into a modified version of pRS416 between HinDIII and Xho I. AclI, SpeI, and BglII sites were introduced into the *MID2* sequence to facilitate cloning of overlapping oligonucleotides encoding polyleucine stretches to replace the Mid2 TMD.

Integration plasmids to express TMD chimeras of Mid2-EGFP under the *GAL1* promoter or *MID2* promoter were constructed as follows: *MID2* promoter-NatMX-*GAL1* promoter-*MID2(chimera)*-EGFP-*MID2* terminator - in pBluescriptII(KS-). Homologous recombination was performed using a unique SnaB1 site in the *MID2* gene for expression from the endogenous promoter, or using the unique sites HpaI and Blp1 for expression from the *GAL1* promoter. All constructs were sequenced. All genetic manipulations were performed using the Traffo method for transforming yeast strains [56], and deletions were verified using check PCR to assay for loss of the endogenous gene copy. For a complete list of primers used see Table S4.

### Criteria for Library Construction

For this work we assembled two “mini libraries” by choosing strains of interest from the above commercially available yeast libraries. First we chose 379 strains that represent a variety of possible cargo (the cargo library) from the GFP library in which each ORF is C-terminally tagged with GFP, thus enabling the visualization of the sub-cellular localization of a protein under control of its own promoter [15]. To assemble the library, we hand-picked all possible cargo proteins—those which had been visualized as being localized to either the plasma membrane, Golgi apparatus, vacuolar membrane, vacuolar lumen, COPI vesicles, COPII vesicles, peroxisomes, adiposomes, or endosomes. In addition, we added all proteins that had an undefined punctate localization (a full list of selected cargo strains is available in Table S5). The initial array was visualized and only strains displaying a strong and correctly localized GFP signal were put into the final array. The second library contained strains mutated in ER to Golgi trafficking proteins (the trafficking library), either from the yeast deletion library that contains deletions of all non-essential proteins [11] or from the DAmP library that contains hypomorphic alleles of the essential ones (for a full list of strains included see Table S6) [24].

### Robotic Library Manipulations

All genetic manipulations were performed using SGA techniques to allow efficient introduction of a trait (mutation or marker) into systematic yeast libraries. SGA was performed as previously described [13],[14],[23],[58]. Briefly, using a RoToR bench-top colony arrayer (Singer Instruments) to manipulate libraries in high-density formats (384 or 1,536), haploid strains from opposing mating types, each harboring a different genomic alteration, were mated on rich media plates. Diploid cells were selected on plates containing all selection markers found on both parent haploid strains. Sporulation was then induced by transferring cells to nitrogen starvation plates. Haploid cells containing all desired mutations were selected for by transferring cells to plates containing all selection markers alongside the toxic amino acid derivatives canavanine and thialysine (Sigma-Aldrich) to select against remaining diploids. Each SGA procedure was validated by inspecting representative strains for the presence of the GFP-tagged cargo and for the correct genotype using check PCR (primer sequences can be found in Table S4).

### High-Throughput Fluorescence Microscopy

Microscopic screening was performed using an automated microscopy set-up as previously described [14]. Briefly, cells were moved from agar plates into liquid 384-well polystyrene growth plates using the RoTor arrayer. Liquid cultures were grown overnight in SD medium, with appropriate auxotrophic selections where applicable, in a shaking incubator (LiCONiC Instruments) in 30°C. A JANUS liquid handler (Perkin Elmer), which is connected to the incubator, was used to back-dilute the strains into plates containing the same medium, after which plates were transferred back to the incubator and were allowed to grow for 3.5 h at 30°C to reach logarithmic growth. The liquid handler was then used to transfer strains into glass bottom 384-well microscope plates (Matrical Bioscience) coated with Concanavalin A (Sigma-Aldrich) to allow formation of a cell monolayer. Wells were washed twice in medium to remove unconnected cells and plates were transferred into an automated inverted fluorescent microscopic ScanR system (Olympus) using a swap robot (Hamilton). The ScanR system is designed to allow auto focus and imaging of plates in 384-well format using a 60× air lens and is equipped with a cooled CCD camera. Images were acquired at excitation at 490/20 nm and emission at 535/50 nm (GFP). After acquisition images were manually reviewed using the ScanR analysis program. Images were processed by the Adobe Photoshop CS3 program for slight contrast and brightness adjustments.

### Manual Fluorescence Microscopy

Manual Microscopy was performed using either one of two systems: for Figures S3 and S4 we used an Olympus IX71 microscope controlled by the Delta Vision SoftWoRx 3.5.1 software with ×100 oil lens. Images were captured by a Phoetometrics Coolsnap HQ camera with excitation at 490/20 nm and emission at 528/38 nm (GFP) or excitation at 555/28 nm and emission at 617/73 nm (mCherry/RFP). Images were transferred to Adobe Photoshop CS3 for slight contrast and brightness adjustments.

For Figures 4–8 we used a 100×1.49 NA objective on a Nikon Eclipse TE2000 epifluorescent microscope using a CCD camera (CoolSNAP-HQ2, Roper Scientific) and RFP and GFP filters (Chroma Technology). Images were acquired and analyzed using MetaMorph and ImageJ, and normalized using Adobe Photoshop. For some co-localization studies with Golgi markers both channels were imaged simultaneously using a beam splitter (Cairn Research). For fusions expressed under the *MID2 p*romoter strains were grown in synthetic complete medium to reduce background fluorescence.

### Protein Purification

For protein purification during the galactose-induced pulse chases we first added 3 OD_600_ of cells to NaN_3_ (*t* = 0). For subsequent time points 1 ml of cells were collected. Cells were resuspended in 500 µl NaOH solution (0.2 M NaOH, 0.2% β-mercaptoethanol) and precipitated in 5% trichloroacetic acid. Pellets were resuspended in sample buffer and 10 µl Tris base. After electrophoresis on 4%–20% gradient gels (Novex, Invitrogen), immunoblots were blotted with mouse anti-GFP (7.1/13.1, Roche), HRP anti-mouse, and ECL (Amersham). For quantitation purposes, gel lane profiles were obtained from scanned autoradiograms and peak areas were determined using ImageJ. The ratio of the ER peak to the sum of the ER, post-ER, and free-GFP peaks was calculated and normalized so that it was 1.0 at the start of the chase (*t* = 0).

## Supporting Information

Figure S1 **PAIRS recapitulates cargo identity of previously studied cargo receptors.** Deletion of Chs7 and Emp24 causes ER retention of previously studied cargo. Shown are control strains (wild type [WT]) relative to Δ*chs7* (A) and Δ*emp24* (B).
(PDF)

Figure S2 **Deletion of Erv14 causes localization changes in a variety of proteins.** (A) Shown are representative images of 23 GFP tagged proteins that were retained in the ER in the absence of Erv14 during logarithmic growth. Shown are control strains (wild type [WT]) relative to Δ*erv14.* (B) Shown are representative images of Golgi proteins that change localization in Δ*erv14* strains and display cytosolic fluorescence during logarithmic growth. Since ER retention is not observed they probably do not represent bona fide cargo. Materials and methods for this figure can be found in accompanying files “Text S1.”
(PDF)

Figure S3 **Erv14 and Erv15 are not required for proper localization of tail-anchored proteins nor glycosylphosphatidylinositol-anchored proteins.** Control and mutant cells were transformed with plasmids driving expression of (A) N-terminally tagged, tail-anchored proteins (photographed at 60×) or (B) GPI-anchored proteins (photographed at 100×). Localization during logarithmic growth was not dependant on either Erv14 or Erv15.
(PDF)

Figure S4 **Analysis of Erv14 interacting proteins validates the genetic predictions.** (A) Cargo proteins revealed by mass spectrometry to bind Erv14. (B) Some of the proteins suggested by the mass spectrometry as possible Erv14 cargo were indeed identified as such upon N-terminally tagging with GFP. Materials and methods for this figure can be found in accompanying files “Text S1.”
(PDF)

Figure S5 **Deletion of** ***erv14*** **slows ER exit of Mep2-GFP.** Yeast cells expressing Mep2-GFP under an inducible (GalS) promoter were visualized for dynamics of ER exit in control (wild type [WT]) and Δ*erv14* cells. Yeast were grown in raffinose-containing media and galactose was added at time 0 from which cells were visualized every 10 min. Time of appearance of ER localization is identical in control and mutant cells and is marked by a red arrow pointing to the cells displaying ER localization. Time of appearance of plasma membrane localized Mep2-GFP differs and is marked by a blue arrow to demonstrate the cells that now have plasma membrane localization. Materials and methods for this figure can be found in accompanying files “Text S1.”
(PDF)

Figure S6 **ER exit rate of TMD variants of Mid2-GFP.** (A) Anti-GFP immunoblots of whole cell lysates from yeast expressing the indicated polyleucine TMD variants of Mid2-GFP (as in Figure 4D). The variants were expressed under the control of the GAL1 promoter from constructs integrated at the MID2 locus. The cells were induced with galactose for 2 h, and then harvested at the times indicated after replacing the medium with that containing 2% glucose. The arrows indicate the ER form (ER) and Golgi modified form (G) of Mid2, and free GFP. (B) As (A), except that the polyleucine TMDs are flanked with tryptophans as indicated in the alignment, and the MID2-GFP variants were expressed under the control of the GAL1 promoter from a centromeric plasmid. The arrows indicate the ER form (ER) and Golgi modified form (G) of Mid2, free GFP, and a clipped form that has been previously seen with Mid2 and is generated in a post-Golgi compartment [37].
(PDF)

Table S1 **ER to Golgi cargo receptors and their cargo.**
(DOCX)

Table S2 **Plasmids used in this study.**
(DOCX)

Table S3 **List of yeast strains used in this study.**
(DOCX)

Table S4 **PCR Primers utilized in the study.**
(DOCX)

Table S5 **List of C-terminal fusion GFP ORFs inspected in this study (see separate file).**
(XLSX)

Table S6 **List of DAmP of genes involved in ER to Golgi trafficking.**
(DOCX)

Text S1 **Supplementary “Materials and Methods” and their references.**
(DOCX)

## Abbreviations

DAmP: decreased abundance by mRNA perturbation
ER: endoplasmic reticulum
GFP: green fluorescent protein
GPI: glycosylphosphatidylinositol
PAIRS: pairing analysis of cargo receptors
SGA: synthetic genetic array
TMD: transmembrane domain
