# Supplementary figures and images for: A Systematic Approach to Pair Secretory Cargo Receptors with Their Cargo Suggests a Mechanism for Cargo Selection by Erv14
Source: PLoS Biol. 2012 May 22;10(5):e1001329. doi: 10.1371/journal.pbio.1001329 (PMC3358343; doi:10.1371/journal.pbio.1001329)

Herzig *et al.*, Supplementary Figure 1

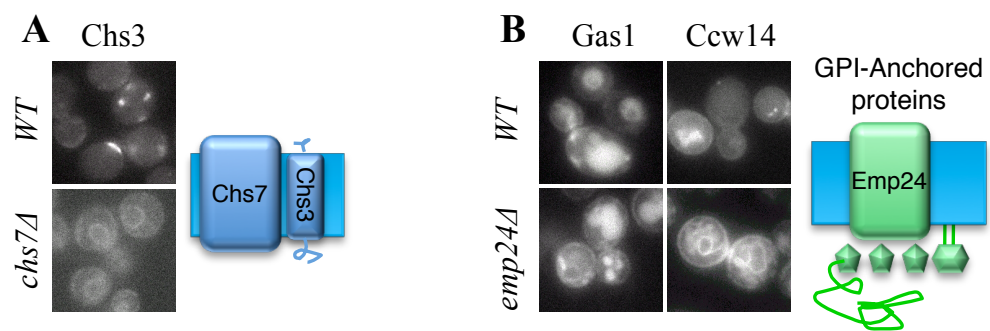

Supplement: Figure S1 — PAIRS recapitulates cargo identity of previously studied cargo receptors. Deletion of Chs7 and Emp24 causes ER retention of previously studied cargo. Shown are control strains (wild type [WT]) relative to Δchs7 (A) and Δemp24 (B). (PDF) [file pbio.1001329.s001.pdf]

Herzig *et al.*, Supplementary Figure 2

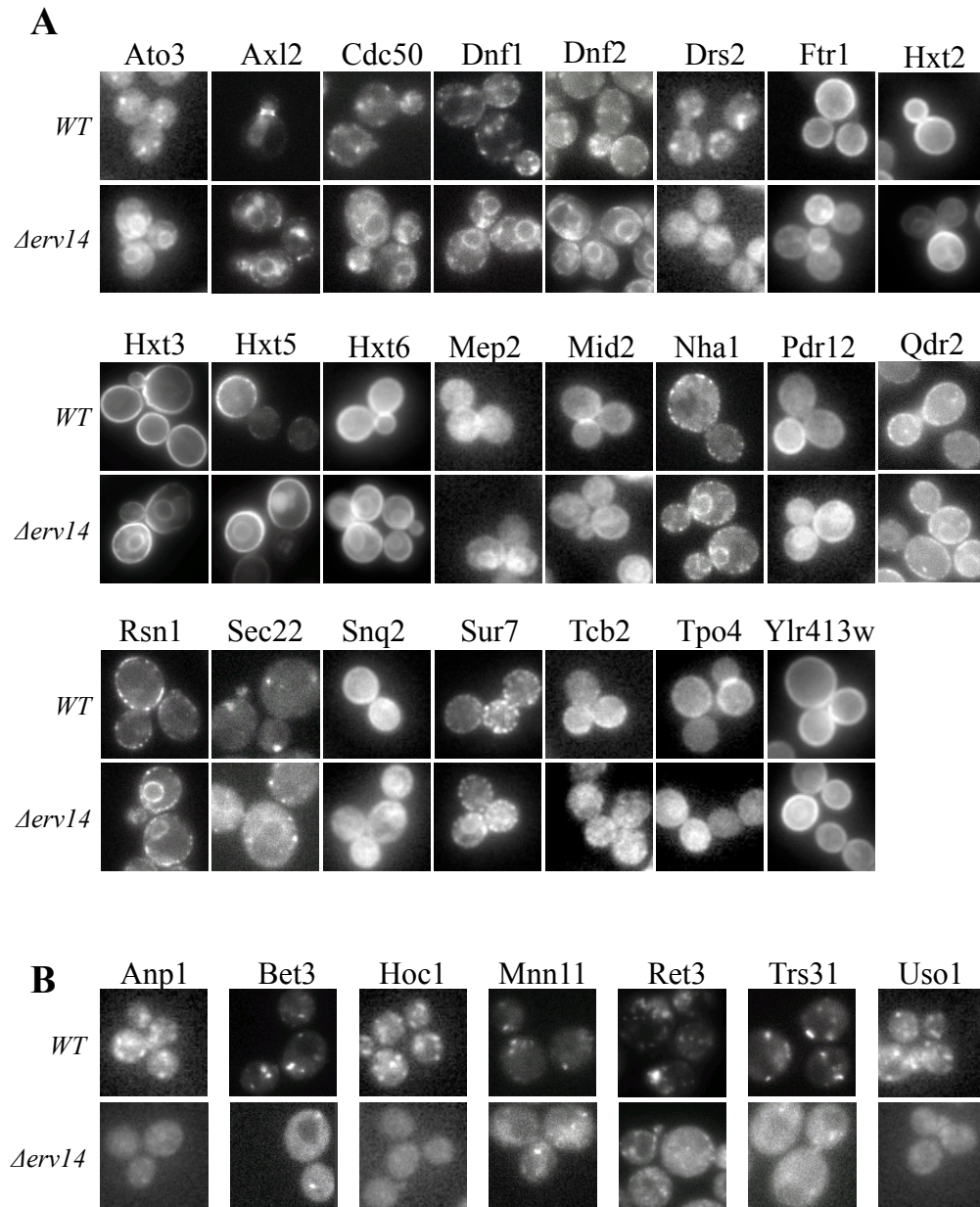

Supplement: Figure S2 — Deletion of Erv14 causes localization changes in a variety of proteins. (A) Shown are representative images of 23 GFP tagged proteins that were retained in the ER in the absence of Erv14 during logarithmic growth. Shown are control strains (wild type [WT]) relative to Δerv14. (B) Shown are representative images of Golgi proteins that change localization in Δerv14 strains and display cytosolic fluorescence during logarithmic growth. Since ER retention is not observed they probably do not represent bona fide cargo. Materials and methods for this figure can be found in accompanying files “Text S1.” (PDF) [file pbio.1001329.s002.pdf]

**A**

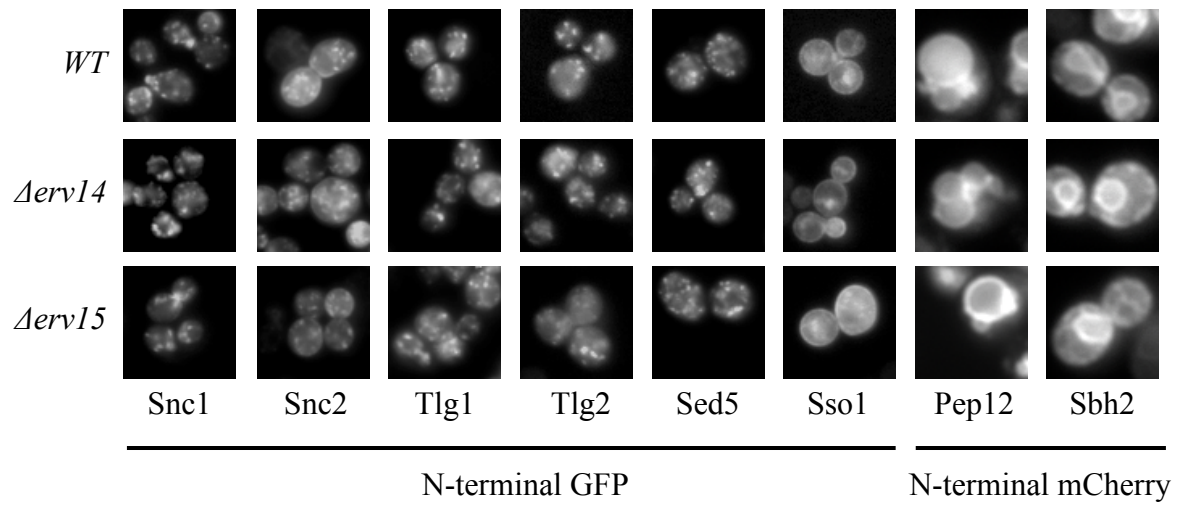

**B**

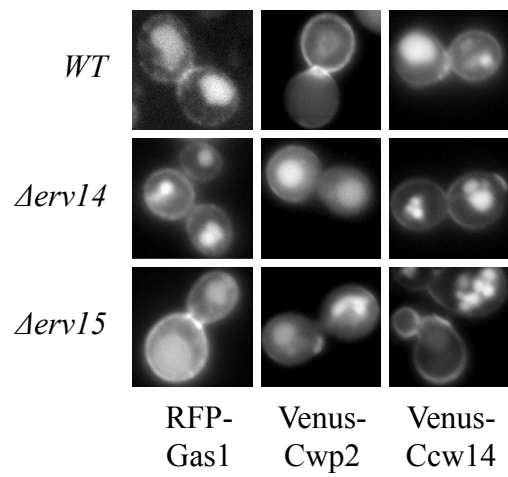

Supplement: Figure S3 — Erv14 and Erv15 are not required for proper localization of tail-anchored proteins nor glycosylphosphatidylinositol-anchored proteins. Control and mutant cells were transformed with plasmids driving expression of (A) N-terminally tagged, tail-anchored proteins (photographed at 60×) or (B) GPI-anchored proteins (photographed at 100×). Localization during logarithmic growth was not dependant on either Erv14 or Erv15. (PDF) [file pbio.1001329.s003.pdf]

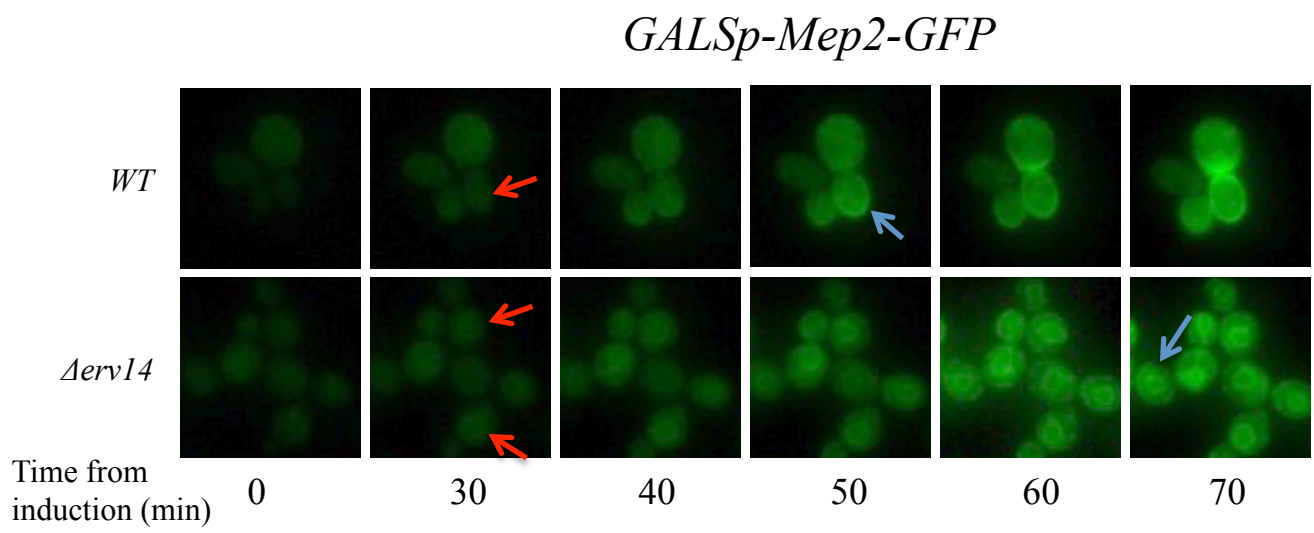

Supplement: Figure S5 — Deletion of erv14 slows ER exit of Mep2-GFP. Yeast cells expressing Mep2-GFP under an inducible (GalS) promoter were visualized for dynamics of ER exit in control (wild type [WT]) and Δerv14 cells. Yeast were grown in raffinose-containing media and galactose was added at time 0 from which cells were visualized every 10 min. Time of appearance of ER localization is identical in control and mutant cells and is marked by a red arrow pointing to the cells displaying ER localization. Time of appearance of plasma membrane localized Mep2-GFP differs and is marked by a blue arrow to demonstrate the cells that now have plasma membrane localization. Materials and methods for this figure can be found in accompanying files “Text S1.” (PDF) [file pbio.1001329.s005.pdf]

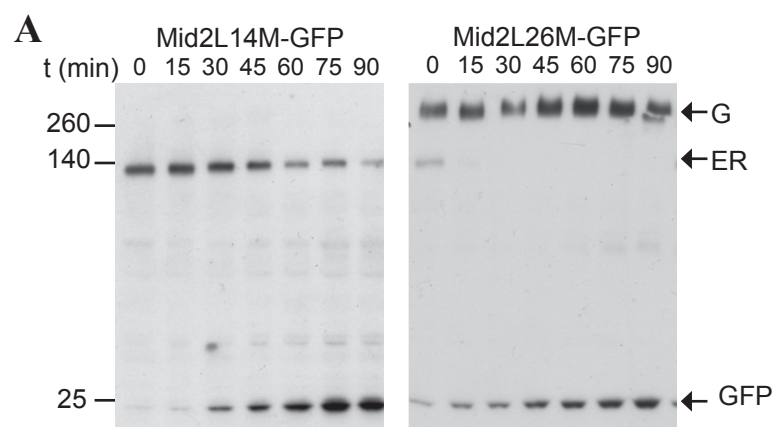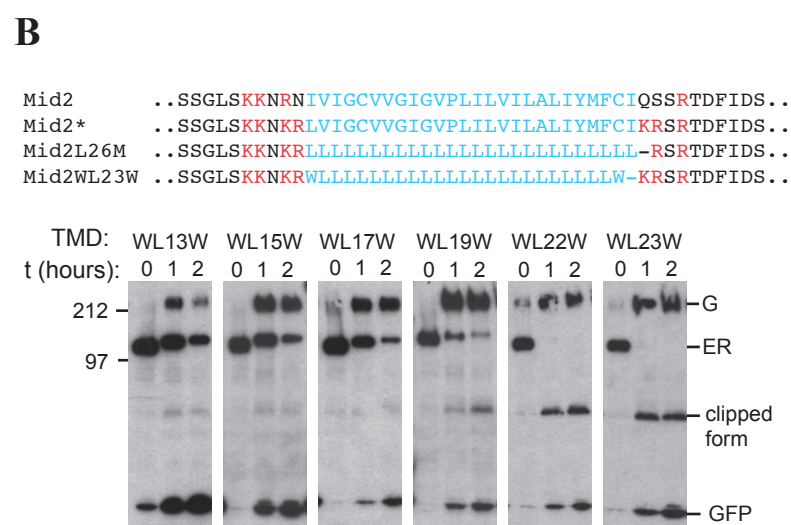

Supplement: Figure S6 — ER exit rate of TMD variants of Mid2-GFP. (A) Anti-GFP immunoblots of whole cell lysates from yeast expressing the indicated polyleucine TMD variants of Mid2-GFP (as in Figure 4D). The variants were expressed under the control of the GAL1 promoter from constructs integrated at the MID2 locus. The cells were induced with galactose for 2 h, and then harvested at the times indicated after replacing the medium with that containing 2% glucose. The arrows indicate the ER form (ER) and Golgi modified form (G) of Mid2, and free GFP. (B) As (A), except that the polyleucine TMDs are flanked with tryptophans as indicated in the alignment, and the MID2-GFP variants were expressed under the control of the GAL1 promoter from a centromeric plasmid. The arrows indicate the ER form (ER) and Golgi modified form (G) of Mid2, free GFP, and a clipped form that has been previously seen with Mid2 and is generated in a post-Golgi compartment [37]. (PDF) [file pbio.1001329.s006.pdf]
