# Supplementary material for: A Systematic Approach to Pair Secretory Cargo Receptors with Their Cargo Suggests a Mechanism for Cargo Selection by Erv14
Source: PLoS Biol. 2012 May 22;10(5):e1001329. doi: 10.1371/journal.pbio.1001329 (PMC3358343; doi:10.1371/journal.pbio.1001329)

Herzig *et al.*, Supplementary Figure 4

A

**Protein name**	**GFP fusion protein display ER retention in *erv14Δ***
Cps1	+
Dnf2	+
Hoc1	+
Hxt1	+
Hxt2	+
Hxt3	+
Hxt4	-
Hxt5	+
Hxt6/7	+
Kre2	-
Ktr1	-
Ktr3	-
Mnn11	+
Mnn5	-
Pdr5	+
Qdr2	+
Ssp120	-
Tcb1	-
Tcb2	+
Tcb3	-
Tna1	+
Van1	-
Vph1	-
Ynl194c	-

B

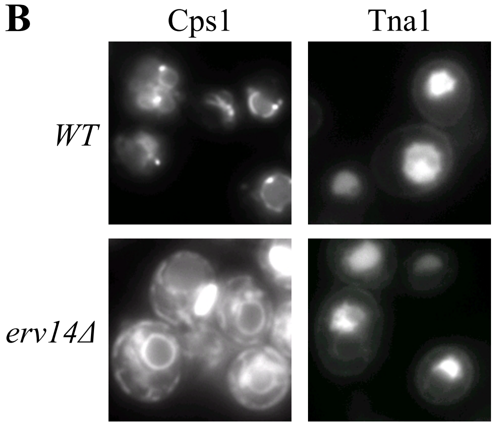

Supplement: Figure S4 — Analysis of Erv14 interacting proteins validates the genetic predictions. (A) Cargo proteins revealed by mass spectrometry to bind Erv14. (B) Some of the proteins suggested by the mass spectrometry as possible Erv14 cargo were indeed identified as such upon N-terminally tagging with GFP. Materials and methods for this figure can be found in accompanying files “Text S1.” (PDF) [file pbio.1001329.s004.pdf]
