# Supplementary material for: A Systematic Approach to Pair Secretory Cargo Receptors with Their Cargo Suggests a Mechanism for Cargo Selection by Erv14
Source: PLoS Biol. 2012 May 22;10(5):e1001329. doi: 10.1371/journal.pbio.1001329 (PMC3358343; doi:10.1371/journal.pbio.1001329)
Supplement: Table S1 — ER to Golgi cargo receptors and their cargo. (DOCX) [file pbio.1001329.s007.docx]

**Supplementary Table I. ER to Golgi CRs and their cargo**

| **Cargo Receptor** | **Suggested Cargo** | **References** |
| --- | --- | --- |
| Erv14 | Axl2  Sma2  Gap1, Hxt1, Hxt2, Mid2 | (Nakanishi *et al*, 2007)  (Powers and Barlowe, 1998, 2002)  (Castillon *et al*, 2009) |
| Erv15 | None suggested |  |
| Erv26 | Pro-Alkaline Phosphatase  Golgi Mannosyltransferases | (Bue *et al*, 2006) (Inadome *et al*, 2005; Noda and Yoda, 2010) |
| Erv29 | CPY, Proteinase A and gpαf | (Belden and Barlowe, 2001; Caldwell *et al*, 2001) |
| Erv41-Erv46 complex | None suggested | (Otte *et al*, 2001) |
| p24 complex (Emp24, Erv25, Erp1, Erp2) | GPI anchored proteins | (Castillon *et al*, 2009; Muniz *et al*, 2000; Schimmoller *et al*, 1995) |
| Emp46/47 | Glycoproteins | (Sato and Nakano, 2002) |
| Shr3 | amino-acid permeases | (Kota *et al*, 2007; Kuehn *et al*, 1996; Ljungdahl *et al*, 1992) |
| Chs7 | Chs3 | (Trilla *et al*, 1999) |
| Gsf2 | Certain sugar permeases (Gal2, Hxt1) | (Sherwood and Carlson, 1999) |
| Pho86 | Pho84 | (Lau *et al*, 2000) |
