# Supplementary material for: A Systematic Approach to Pair Secretory Cargo Receptors with Their Cargo Suggests a Mechanism for Cargo Selection by Erv14
Source: PLoS Biol. 2012 May 22;10(5):e1001329. doi: 10.1371/journal.pbio.1001329 (PMC3358343; doi:10.1371/journal.pbio.1001329)
Supplement: Table S2 — Plasmids used in this study. (DOCX) [file pbio.1001329.s008.docx]

**Supplementary Table II. Plasmids used in this study**

| Name | Source | Utilization |
| --- | --- | --- |
| pRS316 Erv14-HA-3'UTR | (Powers *et al*, 1998) | Expression of Erv14 from its endogenous promoter, C-terminally tagged with HA, followed by its 3’UTR |
| pFA6a-KanMX6 | (Longtine *et al*, 1998) | PCR-based homologous recombination to KO a gene |
| pFA6a-NatMX6 | (Goldstein and McCusker, 1999) |  |
| pYM-N31: natNT2::GalS | (Janke *et al*, 2004) | PCR-based homologous recombination to replace endogenous promoter with *GALSp* |
| pYM-N17: natNT2::*GPDp-GFP* | (Janke *et al*, 2004) | PCR-based homologous recombination to add N-terminal GFP driven by the *GPDp* |
| pRS315 GFP-Sed5 | (Schuldiner *et al*, 2008) | Expression of N-terminal fluorescent marker fused to the ORF of various TA proteins |
| yCP50 GFP-SSO1 | (Gabriely *et al*, 2008) |  |
| yCP50 GFP-SNC1 | (Robinson *et al*, 2006) |  |
| pRS315 GFP-SNC2 | (Robinson *et al*, 2006) |  |
| pAD6 GFP-Tlg1 | (Kama *et al*, 2007) |  |
| pRS315 GFP-Tlg2 | (Kama *et al*, 2007) |  |
| pRS316 Cherry-PEP12 | (Schuldiner *et al*, 2008) |  |
| pRS316 Cherry-SBH2 | (Schuldiner *et al*, 2008) |  |
| pRS416 RFP-Gas1 | Kindly provided by Howard Riezman | Expression of N-terminal fluorescent marker fused to the ORF of various GPI-anchored proteins |
| pRS416 Venus-Cwp2 | (Castillon *et al*, 2009) |  |
| pRS416 Venus-Ccw14 | (Castillon *et al*, 2009) |  |
