# Supplementary material for: A Systematic Approach to Pair Secretory Cargo Receptors with Their Cargo Suggests a Mechanism for Cargo Selection by Erv14
Source: PLoS Biol. 2012 May 22;10(5):e1001329. doi: 10.1371/journal.pbio.1001329 (PMC3358343; doi:10.1371/journal.pbio.1001329)
Supplement: Table S3 — List of yeast strains used in this study. (DOCX) [file pbio.1001329.s009.docx]

**Supplementary Table III. List of yeast strains used in this study**

| name | Mating Type | Genetic Background | Source |
| --- | --- | --- | --- |
| BY4741 | MATa | *his3Δ1 leu2Δ0 met15Δ0 ura3Δ0* | (Brachmann *et al*, 1998) |
| BY4743 | Diploid | *his3Δ1/his3Δ1 leu2Δ0/leu2Δ0 lys2Δ0/+ met15Δ0/+ ura3Δ0* | (Brachmann *et al*, 1998) |
| YMS196 | MATα | *his3∆1 leu2∆0 ura3∆0 cyh2*  *can1∆::STE2pr-spHIS5*  *lyp1∆::STE3pr-LEU2* | sporulated from Strain Y8091 from C. Boone’s lab. |
| YMS721 | MATα | *his3∆1 leu2∆0 met15∆0 ura3∆0*  *can1∆::STE2pr-spHIS5*  *lyp1∆::STE3pr-LEU2* | (Breslow *et al*, 2008) |
| YMS817 | MATα | YMS196 NAT^r^::GalSp-Mep1-GFP::*HIS3* | This study |
| YMS818 | MATα | YMS196 *erv14∆*::KAN^r^ NAT^r^::GalSp-Mep1-GFP::*HIS3* | This study |
| YMS819 | MATα | YMS196 NAT^r^::*GALSp*-Mep2-GFP::*HIS3* | This study |
| YMS820 | MATα | YMS196 erv14∆::KAN^r^ NAT^r^::*GALSp*-Mep2-GFP::*HIS3* | This study |
| YMS792 | MATa | BY4741 *erv14∆*::KAN^r^ | This study |
| YMS793 | MATa | BY4741 *erv15∆*::KAN^r^ | This study |
| YMS954 | MATa | BY4741 *emp24∆*::KAN^r^ | This study |
|  | MATa | BY4741 *Shr3-DAmP* | (Breslow *et al*, 2008) |
|  | MATa | BY4741 *emp47∆*::KAN^r^ | (Giaever *et al*, 2002) |
|  | MATa | BY4741 *erv26∆*::KAN^r^ | (Giaever *et al*, 2002) |
|  | MATa | BY4741 *erv29∆*::KAN^r^ | (Giaever *et al*, 2002) |
|  | MATa | BY4741 *erv41∆*::KAN^r^ | (Giaever *et al*, 2002) |
|  | MATa | BY4741 *chs7∆*::KAN^r^ | (Giaever *et al*, 2002) |
|  | MATa | BY4741 *gsf2∆*::KAN^r^ | (Giaever *et al*, 2002) |
| YMS1001 | MATa | BY4741 NAT^r^::*GPDp*-GFP-Cps1 | This study |
| YMS1005 | MATa | BY4741 NAT^r^::*GPDp*-GFP-Tna1 | This study |
| YMS1006 | MATa | BY4741 *erv14∆*::KAN^r^ NAT^r^::*GPDp*-GFP-Cps1 | This study |
| YMS1010 | MATa | BY4741 *erv14∆*::KAN^r^ NAT^r^::*GPDp*-GFP-Tna1 | This study |
