# Supplementary material for: A Systematic Approach to Pair Secretory Cargo Receptors with Their Cargo Suggests a Mechanism for Cargo Selection by Erv14
Source: PLoS Biol. 2012 May 22;10(5):e1001329. doi: 10.1371/journal.pbio.1001329 (PMC3358343; doi:10.1371/journal.pbio.1001329)
Supplement: Table S4 — PCR Primers utilized in the study. (DOCX) [file pbio.1001329.s010.docx]

**Supplementary Table IV – PCR Primers utilized in the study**

| Primer Name | Sequence | Product Size | Purpose | Template |
| --- | --- | --- | --- | --- |
| ERV14-KO-F | GGTGCTTGGTTATTTATCCTTGCTGTTGTTGTCAACTGTACGGATCCCCGGGTTAATTAA | 1559bp | Primers for deleting *ERV14*'s ORF, leaving just the first 40bp downstream of beginning of ORF | pFA6a-KanMX6 |
| ERV14-KO-R | TGGCCCTTCAGTCTTCTTTGGATTTCAATGTCTTGTTGGAGAATTCGAGCTCGTTTAAAC |  |  |  |
| ERV15-KO-F | CTAACTGCAGGCACCAAGAAGTTCGAAGCCAAGAGTAAAGCGGATCCCCGGGTTAATTAA | 1559bp | Primers for deleting *ERV15*'s ORF, | pFA6a-KanMX6 |
| ERV15-KO-R | GATATGCACAAATCTCTCTTTTTTTTTCCTGTTGTCGCTAGAATTCGAGCTCGTTTAAAC |  |  |  |
| EMP24-KO-F | TTAATAGTATCCCTCCGCACAAAAATACACACGCATAAGGCGGATCCCCGGGTTAATTAA | 1559bp | Primers for deleting *EMP24*'s ORF, | pFA6a-KanMX6 |
| EMP24-KO-R | GCAAAAGTAAATAGATATGAACTACATTTTCCTGCTTTACGAATTCGAGCTCGTTTAAAC |  |  |  |
| Mep2-GalS-F | TATATCATACTTAATATATTACAATACAATATCAACAATGCGTACGCTGCAGGTCGAC | 1775bp | Primers for replacing *MEP2*'s endogenous promoter with the GalS inducible promoter | pYM-N31 |
| Mep2-GalS-R | CCGTTCCTTCGCCTGTAGGCGTACCTGTAAAATTGTAAGACATCGATGAATTCTCTGTCG |  |  |  |
| Cps1-NGFP-F | CATCACATTAAGGAATCATTCATCTAACAATTACATTATGCGTACGCTGCAGGTCGAC | 2743bp | Primers for N-terminally tagging *CPS1* with GFP, expression driven by the GPD constitutive promoter | pYM-N17 |
| Cps1-NGFP-R | ATAGGGACTTTCTAGGGGCCTTCTCTACTGGTAAGGCGATCATCGATGAATTCTCTGTCG |  |  |  |
| Tna1-NGFP-F | TCACTTTCGAGCATTGAACTATTGTCATTACCTCTAGATGCGTACGCTGCAGGTCGAC | 2743bp | Primers for N-terminally tagging *TNA1* with GFP, expression driven by the GPD constitutive promoter | pYM-N17 |
| Tna1-NGFP-R | CCACCAAATGCTTAGGTGACTCCATTGTAAATTTGTTGCTCATCGATGAATTCTCTGTCG |  |  |  |
| ERV14-WTchkORF-F | TTGCATGGTGCTTTGTCATT | 250bp | Primers for checking existence of a WT copy of *ERV14* in the genome | Genome |
| ERV14-WTchkORF-R | CATCACCACTTTCAGCAATCA |  |  |  |
| ERV15-WTchkORF-F | TGTCAGGAACCGGATTATCG | 298bp | Primers for checking existence of a WT copy of *ERV15* in the genome | Genome |
| ERV15-WTchkORF-R | ATATCCGTGGCGTCCAGTAG |  |  |  |
| Gsf2-wtchk-F | TACCGATTTGCCCGACGCCA | 362bp | Primers for checking existence of a WT copy of *GSF2* in the genome | Genome |
| Gst2-wtchk-R | AGCCCAGAGAGCGCAAACGT |  |  |  |
| Erv29-wtchk-F | AGGACCATACTCTGGCGGTGCA | 376bp | Primers for checking existence of a WT copy of *ERV29* in the genome | Genome |
| Erv29-wtchk-R | AGCACATAACACACCGGTGGCA |  |  |  |
| Erv26-wtchk-F | ACACGAGCTAACCCAACAGCCC | 204bp | Primers for checking existence of a WT copy of *ERV26* in the genome | Genome |
| Erv26-wtchk-R | ACGCAGACGTGCTAGTTTCGCT |  |  |  |
| Emp47-wtchk-F | AGCGACATGGGCTGGATTGCT | 288bp | Primers for checking existence of a WT copy of *EMP47* in the genome | Genome |
| Emp47-wtchk-R | ATGCCACCGTCGGTTTGGCC |  |  |  |
| Emp24-wtchk-F | GTTCTTCTCGGCGTCCGCCC | 214bp | Primers for checking existence of a WT copy of *EMP24* in the genome | Genome |
| Emp24-wtchk-R | TTCACCGTGCGACGTGTCCC |  |  |  |
| Chs7-wtchk-F | TCGGTGTACTGCCACGCTGT | 296bp | Primers for checking existence of a WT copy of *CHS7* in the genome | Genome |
| Chs7-wtchk-R | CCAGCAACATGCACCCGCCA |  |  |  |
| S1-Rev | GTCGACCTGCAGCGTACG |  | S1 primer in reverse complement from the pYM family of plasmids. | Genome |
| Mep2-chk-F | GTGTTCATTCCGAGCCACTT | 223bp | Used to checkPCR N-terminal alterations on *MEP2* in combination with S1-Rev | Genome |
| Cps1-chk-F | AGCCTTCCAAGGTCGGGCAA | 146bp | Used to checkPCR N-terminal alterations on *CPS1* in combination with S1-Rev | Genome |
| Tna1-chk-F | ATTTTCCCCCGTGAGACAGCGA | 133bp | Used to checkPCR N-terminal alterations on *TNA1* in combination with S1-Rev | Genome |
| Act1-chk-F | TGTCACCAACTGGGACGATA | 190bp | Primers for checking existence of a WT copy of *act1* in the genome | Genome |
| Act1-chk-R | GGCTTGGATGGAAACGTAGA |  |  |  |
