# Supplementary material for: A Systematic Approach to Pair Secretory Cargo Receptors with Their Cargo Suggests a Mechanism for Cargo Selection by Erv14
Source: PLoS Biol. 2012 May 22;10(5):e1001329. doi: 10.1371/journal.pbio.1001329 (PMC3358343; doi:10.1371/journal.pbio.1001329)
Supplement: Table S6 — List of DAmP of genes involved in ER to Golgi trafficking. (DOCX) [file pbio.1001329.s012.docx]

**Supplementary Table VI. List of Deletions/Hypomorphic Alleles (DAmP) of genes involved in ER to Golgi trafficking**

| **ORF** | **Allele** | **Functional Annotation** |
| --- | --- | --- |
| YIL004C | BET1-DAmP | Type II membrane protein required for vesicular transport between the endoplasmic reticulum and Golgi complex; v-SNARE with similarity to synaptobrevins |
| YKR068C | BET3-DAmP | Hydrophilic protein that acts in conjunction with SNARE proteins in targeting and fusion of ER to Golgi transport vesicles; component of the TRAPP (transport protein particle) complex |
| YPL085W | SEC16-DAmP | COPII vesicle coat protein required for ER transport vesicle budding; Sec16p is bound to the periphery of ER membranes and may act to stabilize initial COPII complexes; interacts with Sec23p, Sec24p and Sec31p |
| YBL050W | SEC17-DAmP | Peripheral membrane protein required for vesicular transport between ER and Golgi, the 'priming' step in homotypic vacuole fusion, and autophagy; stimulates the ATPase activity of Sec18p; has similarity to mammalian alpha-SNAP |
| YBR080C | SEC18-DAmP | ATPase required for vesicular transport between ER and Golgi, the 'priming' step in homotypic vacuole fusion, autophagy, and protein secretion; releases Sec17p from SNAP complexes; has similarity to mammalian NSF |
| YIL109C | SEC24-DAmP | Component of the Sec23p-Sec24p heterodimer of the COPII vesicle coat, required for cargo selection during vesicle formation in ER to Golgi transport; homologous to Sfb2p and Sfb3p |
| YDR238C | SEC26-DAmP | Essential beta-coat protein of the COPI coatomer, involved in ER-to-Golgi protein trafficking and maintenance of normal ER morphology; shares 43% sequence identity with mammalian beta-coat protein (beta-COP) |
| YDL195W | SEC31-DAmP | Component of the Sec13p-Sec31p complex of the COPII vesicle coat, required for vesicle formation in ER to Golgi transport; mutant has increased aneuploidy tolerance |
| YDR170C | SEC7-DAmP | Guanine nucleotide exchange factor (GEF) for ADP ribosylation factors involved in proliferation of the Golgi, intra-Golgi transport and ER-to-Golgi transport; found in the cytoplasm and on Golgi-associated coated vesicles |
| YDR189W | SLY1-DAmP | Hydrophilic protein involved in vesicle trafficking between the ER and Golgi; SM (Sec1/Munc-18) family protein that binds the tSNARE Sed5p and stimulates its assembly into a trans-SNARE membrane-protein complex |
| YMR218C | TRS130-DAmP | One of 10 subunits of the transport protein particle (TRAPP) complex of the cis-Golgi which mediates vesicle docking and fusion; involved in ER to Golgi membrane traffic; mutation activates transcription of OCH1 |
| YBR254C | TRS20-DAmP | One of 10 subunits of the transport protein particle (TRAPP) complex of the cis-Golgi which mediates vesicle docking and fusion; mutations in the human homolog cause the spondyloepiphyseal dysplasia tarda (SEDL) disorder |
| YDR472W | TRS31-DAmP | One of 10 subunits of the transport protein particle (TRAPP) complex of the cis-Golgi which mediates vesicle docking and fusion; involved in endoplasmic reticulum (ER) to Golgi membrane traffic |
| YDL058W | USO1-DAmP | Essential protein involved in the vesicle-mediated ER to Golgi transport step of secretion; binds membranes and functions during vesicle docking to the Golgi; required for assembly of the ER-to-Golgi SNARE complex |
| YGR172C | YIP1-DAmP | Integral membrane protein required for the biogenesis of ER-derived COPII transport vesicles; interacts with Yif1p and Yos1p; localizes to the Golgi, the ER, and COPII vesicles |
| YLR242C | *arv1Δ* | Protein functioning in transport of glycosylphosphatidylinositol intermediates into ER lumen; required for normal intracellular sterol distribution; human ARV1 required for normal cholesterol and bile acid homeostasis; similar to Nup120p |
| YDL099W | *bug1Δ* | Cis-golgi localized protein involved in ER to Golgi transport; forms a complex with the mammalian GRASP65 homolog, Grh1p; mutants are compromised for the fusion of ER-derived vesicles with Golgi membranes |
| YKL179C | *coy1Δ* | Golgi membrane protein with similarity to mammalian CASP; genetic interactions with GOS1 (encoding a Golgi snare protein) suggest a role in Golgi function |
| YGL200C | *emp24Δ* | Integral membrane component of endoplasmic reticulum-derived COPII-coated vesicles, which function in ER to Golgi transport |
| YLR080W | *emp46Δ* | Integral membrane component of endoplasmic reticulum-derived COPII-coated vesicles, which function in ER to Golgi transport |
| YFL048C | *emp47Δ* | Integral membrane component of endoplasmic reticulum-derived COPII-coated vesicles, which function in ER to Golgi transport |
| YAR002C-A | *erp1Δ* | Protein that forms a heterotrimeric complex with Erp2p, Emp24p, and Erv25p; member, along with Emp24p and Erv25p, of the p24 family involved in ER to Golgi transport and localized to COPII-coated vesicles |
| YAL007C | *erp2Δ* | Protein that forms a heterotrimeric complex with Erp1p, Emp24p, and Erv25p; member, along with Emp24p and Erv25p, of the p24 family involved in ER to Golgi transport and localized to COPII-coated vesicles |
| YDL018C | *erp3Δ* | Protein with similarity to Emp24p and Erv25p, member of the p24 family involved in ER to Golgi transport |
| YOR016C | *erp4Δ* | Protein with similarity to Emp24p and Erv25p, member of the p24 family involved in ER to Golgi transport |
| YHR110W | *erp5Δ* | Protein with similarity to Emp24p and Erv25p, member of the p24 family involved in ER to Golgi transport |
| YGL002W | *erp6Δ* | Protein with similarity to Emp24p and Erv25p, member of the p24 family involved in ER to Golgi transport; the authentic, non-tagged protein is detected in highly purified mitochondria in high-throughput studies |
| YGL054C | *erv14Δ* | Protein localized to COPII-coated vesicles, involved in vesicle formation and incorporation of specific secretory cargo; required for the delivery of bud-site selection protein Axl2p to cell surface; related to Drosophila cornichon |
| YGR284C | *erv29Δ* | Protein localized to COPII-coated vesicles, involved in vesicle formation and incorporation of specific secretory cargo |
| YML067C | *erv41Δ* | Protein localized to COPII-coated vesicles, forms a complex with Erv46p; involved in the membrane fusion stage of transport; has homology to human ERGIC2 (PTX1) protein |
| YDL226C | *gcs1Δ* | ADP-ribosylation factor GTPase activating protein (ARF GAP), involved in ER-Golgi transport; shares functional similarity with Glo3p |
| YER122C | *glo3Δ* | ADP-ribosylation factor GTPase activating protein (ARF GAP), involved in ER-Golgi transport; shares functional similarity with Gcs1p |
| YMR292W | *got1Δ* | Homodimeric protein that is packaged into COPII vesicles and cycles between the ER and Golgi; involved in secretory transport but not directly required for aspects of transport assayed in vitro; may influence membrane composition |
| YDR517W | *grh1Δ* | Acetylated, cis-golgi localized protein involved in ER to Golgi transport; homolog of human GRASP65; forms a complex with the coiled-coil protein Bug1p; mutants are compromised for the fusion of ER-derived vesicles with Golgi membranes |
| YDR108W | *gsg1Δ* | Subunit of TRAPPIII (transport protein particle), a multimeric guanine nucleotide-exchange factor for Ypt1p, required for membrane expansion during autophagy and the CVT pathway; directs Ypt1p to the PAS; late post-replication meiotic role |
| YGR166W | *kre11Δ* | Subunit of TRAPPII, a multimeric guanine nucleotide-exchange factor for Ypt1p; involved in intra-Golgi traffic and the retrograde pathway from the endosome to Golgi; role in cell wall beta-glucan biosynthesis and the stress response |
| YJL117W | *pho86Δ* | Endoplasmic reticulum (ER) resident protein required for ER exit of the high-affinity phosphate transporter Pho84p, specifically required for packaging of Pho84p into COPII vesicles |
| YBR005W | *rcr1Δ* | Protein of the ER membrane involved in cell wall chitin deposition; may function in the endosomal-vacuolar trafficking pathway, helping determine whether plasma membrane proteins are degraded or routed to the plasma membrane |
| YKL212W | *sac1Δ* | Phosphatidylinositol phosphate (PtdInsP) phosphatase involved in hydrolysis of PtdIns[4]P; transmembrane protein localizes to ER and Golgi; involved in protein trafficking and processing, secretion, and cell wall maintenance |
| YLR268W | *sec22Δ* | R-SNARE protein; assembles into SNARE complex with Bet1p, Bos1p and Sed5p; cycles between the ER and Golgi complex; involved in anterograde and retrograde transport between the ER and Golgi; synaptobrevin homolog |
| YOR307C | *sly41Δ* | Protein involved in ER-to-Golgi transport |
| YBR172C | *smy2Δ* | Protein of unknown function involved in COPII vesicle formation; interacts with the Sec23p/Sec24p subcomplex; overexpression suppresses the temperature sensitivity of a myo2 mutant; has similarity to S. pombe Mpd2 |
| YHR181W | *svp26Δ* | Integral membrane protein of the early Golgi apparatus and endoplasmic reticulum, involved in COP II vesicle transport; may also function to promote retention of proteins in the early Golgi compartment |
| YPR095C | *syt1Δ* | Guanine nucleotide exchange factor (GEF) for Arf proteins; promotes activation of Arl1p, which recruits Imh1p to the Golgi; involved in vesicular transport; member of the Sec7-domain family; contains a PH domain |
| YER151C | *ubp3Δ* | Ubiquitin-specific protease that interacts with Bre5p to co-regulate anterograde and retrograde transport between endoplasmic reticulum and Golgi compartments; inhibitor of gene silencing; cleaves ubiquitin fusions but not polyubiquitin |
| YML097C | *vps9Δ* | A guanine nucleotide exchange factor involved in vesicle-mediated vacuolar protein transport; specifically stimulates the intrinsic guanine nucleotide exchange activity of Vps21p/Rab5: similar to mammalian ras inhibitors; binds ubiquitin |
| YNL044W | *yup3Δ* | Protein localized to COPII vesicles, proposed to be involved in ER to Golgi transport; interacts with members of the Rab GTPase family and Yip1p; also interacts with Rtn1p |
| YPR028W | *yop1Δ* | Membrane protein that interacts with Yip1p to mediate membrane traffic; interacts with Sey1p to maintain ER morphology; overexpression leads to cell death and accumulation of internal cell membranes |
