# Supplementary material for: A Systematic Approach to Pair Secretory Cargo Receptors with Their Cargo Suggests a Mechanism for Cargo Selection by Erv14
Source: PLoS Biol. 2012 May 22;10(5):e1001329. doi: 10.1371/journal.pbio.1001329 (PMC3358343; doi:10.1371/journal.pbio.1001329)
Supplement: Text S1 — Supplementary “Materials and Methods” and their references. (DOCX) [file pbio.1001329.s013.docx]

**Supplementary Experimental Procedures**

**Additional yeast strains**

YMS819 and YMS820 were created using a strain expressing Mep2 fused to GFP (taken from the GFP library) either in *WT* or *Δerv14* backgrounds and replacing its endogenous promoter with the *GALS* galactose-induced promoter (a weaker derivative of the *GAL1* promoter (Mumberg *et al*, 1994)), using pYM-N31 (Janke *et al*, 2004) as a template. YMS1001, YMS1005, YMS1006 and YMS1010 were created by N-terminally fusing GFP, whose expression is driven by the *GPD* promoter, to the ORF of *CPS1* or *TNA1*, both in *WT* or *Δerv14* (YMS792) background, with pYM-N17 (Janke *et al*, 2004) as a template. To check for N-terminal alterations, a primer upstream of the ATG was used in combination with a reverse complement primer of the first 20 bases in the recombination amplicon. For a complete list of primers used see Supplementary Table III.

**Immunoprecipitation and Mass Spectrometry Experiments**

For immunoprecipitation of Erv14-HA, microsome volume corresponding to 500μg total membrane protein was solubilized in 200 μl 15mM Tris, pH 7.5, 50mM NaCl, 1mM PMSF, 2% digitonin at 4°C for 30 min followed by centrifugation at 10,000g for 5 min at 4°C to remove unsolubilized material. Solubilized material was immunoprecipitated by transferring supernatant to a fresh tube containing 50 μl anti-HA agarose beads (Sigma-Aldrich) pre-washed three times in same buffer. After binding for 60 min at 4°C, beads with bound protein were washed a total of three times with 800 μl of same buffer without Digitonin. Finally, bound protein was released from beads by addition of 50 μl of same buffer with 250 μg/ml HA peptide (Sigma-Aldrich) followed by shaking 15 min 1400rpm at 37°c. Eluate was then loaded and separated on a 16% Tris-Tricine polyacrylamide gel. Whole lanes were subsequently sent for Mass Spectrometry analysis, where proteins were Trypsinized and analyzed by LC-MS/MS on the Orbitrap (Thermo) mass spectrometer and identified by Sequest 3.31 software against the yeast part of the nr database.

**References to Supplementary Materials**

Belden WJ, Barlowe C (2001) Role of Erv29p in collecting soluble secretory proteins into ER-derived transport vesicles. *Science* **294:** 1528-1531.

Brachmann CB, Davies A, Cost GJ, Caputo E, Li JC, Hieter P, Boeke JD (1998) Designer deletion strains derived from Saccharomyces cerevisiae S288C: a useful set of strains and plasmids for PCR-mediated gene disruption and other applications. *Yeast* **14:** 115-132.

Breslow DK, Cameron DM, Collins SR, Schuldiner M, Stewart-Ornstein J, Newman HW, Braun S, Madhani HD, Krogan NJ, Weissman JS (2008) A comprehensive strategy enabling high-resolution functional analysis of the yeast genome. *Nat Methods* **5:** 711-718.

Bue CA, Bentivoglio CM, Barlowe C (2006) Erv26p directs pro-alkaline phosphatase into endoplasmic reticulum-derived coat protein complex II transport vesicles. *Mol Biol Cell* **17:** 4780-4789.

Caldwell SR, Hill KJ, Cooper AA (2001) Degradation of endoplasmic reticulum (ER) quality control substrates requires transport between the ER and Golgi. *J Biol Chem* **276:** 23296-23303.

Castillon GA, Watanabe R, Taylor M, Schwabe TME, Riezman H (2009) Concentration of GPI-Anchored Proteins upon ER Exit in Yeast. *Traffic* **10:** 186-200.

Gabriely G, Kama R, Gelin-Licht R, Gerst JE (2008) Different domains of the UBL-UBA ubiquitin receptor, Ddi1/Vsm1, are involved in its multiple cellular roles. *Mol Biol Cell* **19:** 3625-3637.

Giaever G, Chu AM, Ni L, Connelly C, Riles L, Veronneau S, Dow S, Lucau-Danila A, Anderson K, Andre B, Arkin AP, Astromoff A, El Bakkoury M, Bangham R, Benito R, Brachat S, Campanaro S, Curtiss M, Davis K, Deutschbauer A, Entian KD, Flaherty P, Foury F, Garfinkel DJ, Gerstein M, Gotte D, Guldener U, Hegemann JH, Hempel S, Herman Z, Jaramillo DF, Kelly DE, Kelly SL, Kotter P, LaBonte D, Lamb DC, Lan N, Liang H, Liao H, Liu L, Luo CY, Lussier M, Mao R, Menard P, Ooi SL, Revuelta JL, Roberts CJ, Rose M, Ross-Macdonald P, Scherens B, Schimmack G, Shafer B, Shoemaker DD, Sookhai-Mahadeo S, Storms RK, Strathern JN, Valle G, Voet M, Volckaert G, Wang CY, Ward TR, Wilhelmy J, Winzeler EA, Yang YH, Yen G, Youngman E, Yu KX, Bussey H, Boeke JD, Snyder M, Philippsen P, Davis RW, Johnston M (2002) Functional profiling of the Saccharomyces cerevisiae genome. *Nature* **418:** 387-391.

Goldstein AL, McCusker JH (1999) Three new dominant drug resistance cassettes for gene disruption in Saccharomyces cerevisiae. *Yeast* **15:** 1541-1553.

Inadome H, Noda Y, Adachi H, Yoda K (2005) Immunoisolaton of the yeast Golgi subcompartments and characterization of a novel membrane protein, Svp26, discovered in the Sed5-containing compartments. *Mol Cell Biol* **25:** 7696-7710.

Janke C, Magiera MM, Rathfelder N, Taxis C, Reber S, Maekawa H, Moreno-Borchart A, Doenges G, Schwob E, Schiebel E, Knop M (2004) A versatile toolbox for PCR-based tagging of yeast genes: new fluorescent proteins, more markers and promoter substitution cassettes. *Yeast* **21:** 947-962.

Kama R, Robinson M, Gerst JE (2007) Btn2, a Hook1 ortholog and potential Batten disease-related protein, mediates late endosome-Golgi protein sorting in yeast. *Mol Cell Biol* **27:** 605-621.

Kota J, Gilstring CF, Ljungdahl PO (2007) Membrane chaperone Shr3 assists in folding amino acid permeases preventing precocious ERAD. *Journal of Cell Biology* **176:** 617-628.

Kuehn MJ, Schekman R, Ljungdahl PO (1996) Amino acid permeases require COPII components and the ER resident membrane protein Shr3p for packaging into transport vesicles in vitro. *Journal of Cell Biology* **135:** 585-595.

Lau WTW, Howson RW, Malkus P, Schekman R, O'Shea EK (2000) Pho86p, an endoplasmic reticulum (ER) resident protein in Saccharomyces cerevisiae, is required for ER exit of the high-affinity phosphate transporter Pho84p. *Proc Natl Acad Sci U S A* **97:** 1107-1112.

Ljungdahl PO, Gimeno CJ, Styles CA, Fink GR (1992) SHR3 - A Novel Component of the Secretory Pathway Specifically Required for Localization of Amino-Acid Permeases in Yeast. *Cell* **71:** 463-478.

Longtine MS, McKenzie A, Demarini DJ, Shah NG, Wach A, Brachat A, Philippsen P, Pringle JR (1998) Additional modules for versatile and economical PCR-based gene deletion and modification in Saccharomyces cerevisiae. *Yeast* **14:** 953-961.

Mumberg D, Muller R, Funk M (1994) Regulatable Promoters of Saccharomyces-Cerevisiae - Comparison of Transcriptional Activity and their Use for Heterologous Expression. *Nucleic Acids Res* **22:** 5767-5768.

Muniz M, Nuoffer C, Hauri HP, Riezman H (2000) The Emp24 complex recruits a specific cargo molecule into endoplasmic reticulum-derived vesicles. *Journal of Cell Biology* **148:** 925-930.

Nakanishi H, Suda Y, Neiman AM (2007) Erv14 family cargo receptors are necessary for ER exit during sporulation in Saccharomyces cerevisiae. *Journal of Cell Science* **120:** 908-916.

Noda Y, Yoda K (2010) Svp26 Facilitates Endoplasmic Reticulum to Golgi Transport of a Set of Mannosyltransferases in Saccharomyces cerevisiae. *J Biol Chem* **285:** 15420-15429.

Otte S, Belden WJ, Heidtman M, Liu J, Jensen ON, Barlowe C (2001) Erv41p and Erv46p: New components of COPII vesicles involved in transport between the ER and Golgi complex. *Journal of Cell Biology* **152:** 503-517.

Powers J, Barlowe C (1998) Transport of Axl2p depends on Erv14p, an ER-vesicle protein related to the Drosophila cornichon gene product. *Journal of Cell Biology* **142:** 1209-1222.

Powers J, Barlowe C (2002) Erv14p directs a transmembrane secretory protein into COPII-coated transport vesicles. *Mol Biol Cell* **13:** 880-891.

Robinson M, Poon PP, Schindler C, Murray LE, Kama R, Gabriely G, Singer RA, Spang A, Johnston GC, Gerst JE (2006) The Gcs1 Arf-GAP mediates Snc1,2 v-SNARE retrieval to the Golgi in yeast. *Mol Biol Cell* **17:** 1845-1858.

Sato K, Nakano A (2002) Emp47p and its close homolog Emp46p have a tyrosine-containing endoplasmic reticulum exit signal and function in glycoprotein secretion in Saccharomyces cerevisiae. *Mol Biol Cell* **13:** 2518-2532.

Schimmoller F, Singerkruger B, Schroder S, Kruger U, Barlowe C, Riezman H (1995) The Absence of Emp24p, A Component of ER-Derived COPII-Coated Vesicles, Causes a Defect in Transport of Selected Proteins to the Golgi. *Embo Journal* **14:** 1329-1339.

Schuldiner M, Metz J, Schmid V, Denic V, Rakwalska M, Schmitt HD, Schwappach B, Weissman JS (2008) The GET complex mediates insertion of tail-anchored proteins into the ER membrane. *Cell* **134:** 634-645.

Sherwood PW, Carlson M (1999) Efficient export of the glucose transporter Hxt1p from the endoplasmic reticulum requires Gsf2p. *Proc Natl Acad Sci U S A* **96:** 7415-7420.

Trilla JA, Duran A, Roncero C (1999) Chs7p, a new protein involved in the control of protein export from the endoplasmic reticulum that is specifically engaged in the regulation of chitin synthesis in Saccharomyces cerevisiae. *Journal of Cell Biology* **145:** 1153-1163.
